# Point-of-caRE DiagnostICs for respiraTOry tRact infectionS (PREDICTORS) study: developing guidance for using C-reactive protein point-of-care tests in the management of lower respiratory tract infections in primary care using a Delphi consensus technique

**DOI:** 10.1136/bmjopen-2025-101438

**Published:** 2025-05-27

**Authors:** Joseph O’Shea, Carmel Hughes, Gerard J Molloy, Cathal Cadogan, Akke Vellinga, Tom Fahey, Cristín Ryan

**Affiliations:** 1 School of Pharmacy and Pharmaceutical Sciences, Trinity College Dublin, Dublin, Ireland; 2 School of Pharmacy, Queen’s University Belfast, Belfast, UK; 3 School of Psychology, University of Galway, Galway, Ireland; 4 School of Public Health, Physiotherapy and Sports Science, University College Dublin, Dublin, Ireland; 5 Department of General Practice, RCSI University of Medicine and Health Sciences, Dublin, Ireland

**Keywords:** Primary Care, Respiratory Therapy, Antibiotics

## Abstract

**Abstract:**

**Objective:**

Antimicrobial resistance is a significant global health challenge, exacerbated by unnecessary antibiotic prescribing. Respiratory tract infections (RTIs) are common reasons for antibiotic prescribing in primary care, despite most being viral or bacterial infections that are self-limiting. C-reactive protein (CRP) point-of-care tests (POCTs) are promising tools to support antibiotic stewardship by guiding the management of lower RTIs (LRTIs). The aim of this study was to develop best practice guidance for using CRP POCT in the management of LRTIs in primary care.

**Design:**

Scoping review findings informed guidance statements, which were then evaluated through a three-round Delphi process with an expert panel via web-based questionnaires. Statements focused on intended use, detection of bacterial LRTIs, communication strategies, device features, performance and ease of use of CRP POCT.

**Setting and participants:**

The panel of experts included 19 healthcare professionals across several specialties, including general practitioners, community pharmacists, hospital pharmacists and respiratory physicians.

**Main outcome measures:**

Panellists rated each guidance statement using a 5-point Likert scale, with acceptance, revision or rejection determined using predefined cut-off scores for medians and interquartile ranges. Statements were revised between rounds using the feedback provided by panellists.

**Results:**

In the first round, 49 statements were evaluated; 16 were accepted, nine removed and 24 revised for the second round. Of the 24 statements evaluated in the second round, 17 were accepted and seven were revised. In the third round, consensus was reached on four of the seven statements presented, resulting in 37 final guidance statements. These statements covered key areas, including the appropriate use of CRP POCTs to guide antibiotic prescribing, CRP cut-off values, integration with clinical decision rules, device performance and operational considerations, training requirements and financial reimbursement. The panel emphasised the need for structured guidelines to align CRP POCT use with clinical context and highlighted its role in improving diagnostic confidence while supporting antibiotic stewardship.

**Conclusions:**

This study provides a set of best practice guidance statements to support the use of CRP POCT in the management of LRTIs in primary care. Dissemination and further research are required to assess their impact.

**STRENGTHS AND LIMITATIONS OF THIS STUDY**

- Rigorous evidence review process: a scoping review, involving a comprehensive, systematic search across multiple databases was undertaken to inform the guidance statements, ensuring they were grounded in peer-reviewed evidence.
- Diverse expert panel: the Delphi panel included a range of clinical experts, including general practitioners, pharmacists practising in the community and hospital setting and respiratory physicians, providing multifaceted insights from the multidisciplinary team that cares for patients with lower respiratory tract infections.
- Structured and transparent Delphi methodology: the predefined criteria for acceptance, rejection or revision of statements, along with the iterative rounds, ensured a systematic approach to achieving consensus, reflecting established best practices in Delphi studies.
- Framework-driven statement development: the draft guidance statements were structured using established criteria (eg, ASSURED), ensuring a comprehensive and methodologically sound approach to defining best practices for C-reactive protein point-of-care test use in primary care.
- Limited generalisability: the study only included participants from Ireland, which may limit the external validity of the findings to other countries or healthcare systems, particularly those with different resources or healthcare structures.

## Introduction

 Antimicrobial resistance (AMR)—the ability of microorganisms to resist treatment with antimicrobials, including antibiotic, antiviral, antifungal and antiparasitic products—is a major global public health threat, undermining the effectiveness of medical treatments.^1^ However, within this broader issue, antibiotic resistance is of particular concern, as excessive antibiotic consumption accelerates the emergence of resistant bacterial infections.^2 3^ In 2021, Naghavi *et al* estimated that bacterial AMR was responsible for 4.71 million deaths globally, with 1.14 million of those deaths directly attributed to resistance. Projections suggest that by 2050, antibiotic resistance could be responsible for 1.91 million deaths and 8.22 million associated deaths worldwide.^4^

In Ireland, antibiotic consumption in primary care remains higher than in many other European countries.^5^ Data from 29 European Union (EU)/European Economic Area nations in 2022 showed that 45% reported primary care antibiotic consumption exceeding the EU average.^5^ Ireland, alongside Bulgaria, Croatia, Italy, Malta, Romania and Slovakia, saw an increase in antibiotic use in 2022 compared with 2019.^5^ This trend reflects broader challenges in antibiotic stewardship across the region. Given the EU’s target of reducing antibiotic use by 20% by 2030,^6^ greater efforts are needed to curb consumption and align Ireland with best practices in antibiotic stewardship.

Respiratory tract infections (RTIs) are the leading indication for antibiotic prescriptions in primary care, accounting for up to 65% of all such prescriptions in Irish general practices.^7^ Despite many RTIs being viral in origin,^8^ unnecessary antibiotic prescribing remains widespread.^9^,^11^ This practice not only fails to target the cause of infection but also reinforces diagnostic uncertainty. Distinguishing between viral and bacterial infections remains a key challenge that clinicians are required to contend with. The frequent use of antibiotics in cases where they are not needed complicates efforts to improve antibiotic prescribing practices.^12 13^

The COVID-19 pandemic significantly reshaped healthcare delivery, prompting innovation and adaptation across health professions.^14 15^ Before the pandemic, antibiotics were prescribed for RTIs in 54% of consultations. This fell to 23% in 2020 and 21% in 2021, driven by changes in healthcare practices, patient behaviours and public health campaigns.^15^ These shifts present an opportunity to build on pandemic-era strategies to sustain more judicious antibiotic use for RTIs and strengthen antibiotic stewardship in primary care settings.

Point-of-care tests (POCTs), also known as near-patient tests (‘laboratory diagnostic testing, performed at or near the site where clinical care is delivered’^16 17^), offer a strategy to support antibiotic prescribing by rapidly distinguishing between bacterial and viral infections.^18^,^22^ Public acceptance of POCTs grew during the COVID-19 pandemic, as widespread use of rapid antigen detection tests for COVID-19 normalised their role in healthcare.^23 24^ Other POCTs, such as rapid antigen detection tests for Group A *Streptococcus,*^25 26^ procalcitonin for sepsis and bacterial RTI diagnosis^27^,^29^ and C-reactive protein (CRP) POCTs, have shown potential in reducing unnecessary antibiotic prescriptions in primary care. CRP POCTs, in particular, are now widely used in countries such as Denmark, Norway and the Netherlands to guide antibiotic use in lower RTIs (LRTIs).^29^,^33^

There is a strong interest in the use of POCTs in primary care, as highlighted by an international survey of 2770 general practitioners (GPs) in the UK, Australia, Belgium, the Netherlands and the USA.^34^ GPs expressed a specific desire for POCTs to aid in diagnosing infections (eg, CRP and chlamydia), cardiac conditions (eg, troponin and B-type natriuretic peptide) and thrombotic disorders (eg, D-dimer).^34^ Varzgaliene *et al* found that Irish GPs shared these opinions, with 70% of survey respondents indicating they would use CRP POCTs if they were available in their practices.^35^ However, for POCTs to be effectively integrated into primary care, they must deliver accurate, reliable, timely and cost-effective results.^36^ Furthermore, a European expert panel has endorsed CRP POCTs as a tool to combat unnecessary antibiotic use for LRTIs but emphasised the need for comprehensive guidelines, reimbursement strategies and educational tools to facilitate access and uptake.^32^ In light of this gap, the present study—conducted as part of the Point-of-caRE DiagnostICs for respiraTOry tRact infectionS (PREDICTORS) study—aimed to develop and reach consensus on guidance statements for the use of CRP POCTs in the management of LRTIs in primary care, using the Delphi consensus technique. Further details on the broader study objectives and work packages can be found in the published PREDICTORS study protocol.^37^

## Methods

### Study design

To develop best practice guidance for using POCTs in the management of RTIs in primary care, a scoping review was conducted following an established protocol to ensure comprehensive literature coverage.^38^ Databases, including PubMed, EMBASE, Web of Science, Cochrane Library, were systematically searched using terms such as ‘primary care’, ‘point-of-care’ and ‘respiratory tract infection’. Additional resources, including the RTI POCT’s manufacturers’ literature and policy documents, were also reviewed. Two reviewers independently screened search results for eligibility and extracted data using a predefined data extraction form. Key data included the type of POCT used, the provider and setting of the test, how and when test results were communicated within the primary healthcare team and the impact of the POCT on antibiotic prescribing.

Following the review of the literature and additional resources and based on the extracted data, the research team developed a draft set of guidance statements for using CRP POCT. Members of the research team had previous experience designing and conducting Delphi studies, which informed the development and conduct of the present study. These statements were then refined and validated through the Delphi consensus technique, a well-established method for achieving expert agreement to support evidence-based decision-making.^39^,^41^ This study was conducted and reported in accordance with the DELPHI-STAR reporting guidelines for Delphi studies in health research.^42^ A completed DELPHI-STAR checklist is provided in online supplemental file 1.

### Compilation of draft best practice guidance statements

The draft statements focused on areas where there was both clear evidence and variation in approaches to CRP testing, aligning with the components of the ASSURED criteria (box 1).^43^ The ASSURED criteria were originally developed for POCTs in diagnosing sexually transmitted infections and have been used to inform diagnostic strategies for urinary tract infections.^44^ They recommend that POCTs should be *A*ffordable, *S*ensitive, *S*pecific, *U*ser-friendly, *R*apid, *E*quipment-free and *D*eliverable to end-users.^43^ The draft statements aligned with the ASSURED criteria, for example, CRP cut-off values and interpretation (Sensitive and Specific), training and communication skills (User-friendly), test turnaround and portability (Rapid/Equipment-free) and funding and policy incentives (Affordable). These were organised into five overarching sections: POCT use; detection of bacterial LRTIs and antibiotic prescribing; communication strategies for antibiotic stewardship; device features and performance; and POCT user operation.

Box 1 **The ASSURED criteria**

- Affordable: to those at risk of infection
- Sensitive: reduce false negative results
- Specific: reduce false positive results
- User-friendly: minimal steps and use of non-invasive specimens
- Rapid and robust: short turnaround time and room temperature storage conditions
- Equipment-free: minimal equipment required to ensure ease of use
- Delivered: accessible to end-users

### Selection of the Delphi panel

To ensure the validity of the consensus process, it was important to maintain participation within the recommended range of 5–25 individuals for achieving consensus in later rounds.^44^,^50^ This approach was supported by recruiting a panel of 25 experts from the research team’s professional networks to ensure diverse stakeholder representation and to mitigate attrition, which is a common challenge in Delphi studies.^51^ The panel included GPs, community pharmacists, hospital pharmacists and respiratory physicians from various regions in Ireland. The Health Service Executive system, which is responsible for delivering public health and social services in Ireland, was represented in this study through its Antimicrobial Resistance & Infection Control (AMRIC) team. The AMRIC team leads a key patient safety programme focused on improving antimicrobial stewardship across both acute and community healthcare settings. Additionally, the Pharmacists Antimicrobial Stewardship Network, a voluntary specialist interest group of pharmacists dedicated to promote responsible antimicrobial use and limit the emergence of AMR, was also represented.

### Patient and public involvement

While patient and public involvement (PPI) is a critical aspect of healthcare research, patients were not included in the Delphi panel as the study aimed to develop and reach consensus on best practice guidance for CRP POCT use by healthcare professionals in primary care. Given that the implementation of CRP POCT is a clinical decision made by prescribers and other healthcare providers, the statements were designed to address diagnostic, operational and clinical decision-making aspects requiring expert input. As a result, the language used in the statements is technical and not necessarily patient-friendly. However, to ensure that patient perspectives were considered, a PPI panel reviewed the draft guidance statements and provided feedback on aspects related to patient experience and communication, which were incorporated into the final recommendations where relevant.

### Data collection

The Delphi process involved three rounds of web-based (Qualtrics) questionnaires (onlinesupplemental files 2^4^).^52^ Informed consent was obtained at the beginning of the questionnaire, and reasons for non-participation were not recorded. Prior to the first round, the questionnaire was piloted by three pharmacists from the School of Pharmacy and Pharmaceutical Sciences, Trinity College Dublin, to assess usability and readability, with revisions made based on their feedback. Questionnaires were distributed in April–June, July–August and September–October 2024, via email, with a link to the questionnaire. Reminder emails were sent at 1 week and 2 weeks after the initial invitation. Panellists rated their agreement with each statement on a 5-point Likert scale (1=strongly disagree, 5=strongly agree)^53^ and were encouraged to provide comments for each statement in rounds 1 and 2.

### Data analysis

Agreement for each statement was assessed using the median response and interquartile range (IQR). Statements were accepted if the lower quartile was ≥4 and rejected if the upper quartile was ≤2. For accepted statements, panel feedback was reviewed and minor wording adjustments were made where necessary to enhance clarity. Statements with an IQR including 3 were reviewed by the research team alongside panel feedback and were either revised for the next round or excluded. In the second round, all revised statements—including those that had been accepted but reworded based on panel feedback and those requiring revision due to an IQR including 3—were redistributed to panel members, along with their individual and group responses. The median and IQR were recalculated and feedback was re-evaluated. Statements that did not achieve consensus in the second round proceeded to a third round. Any statements failing to reach consensus by the final round were ultimately rejected. 

## Results

### Scoping review

**Figure 1:**
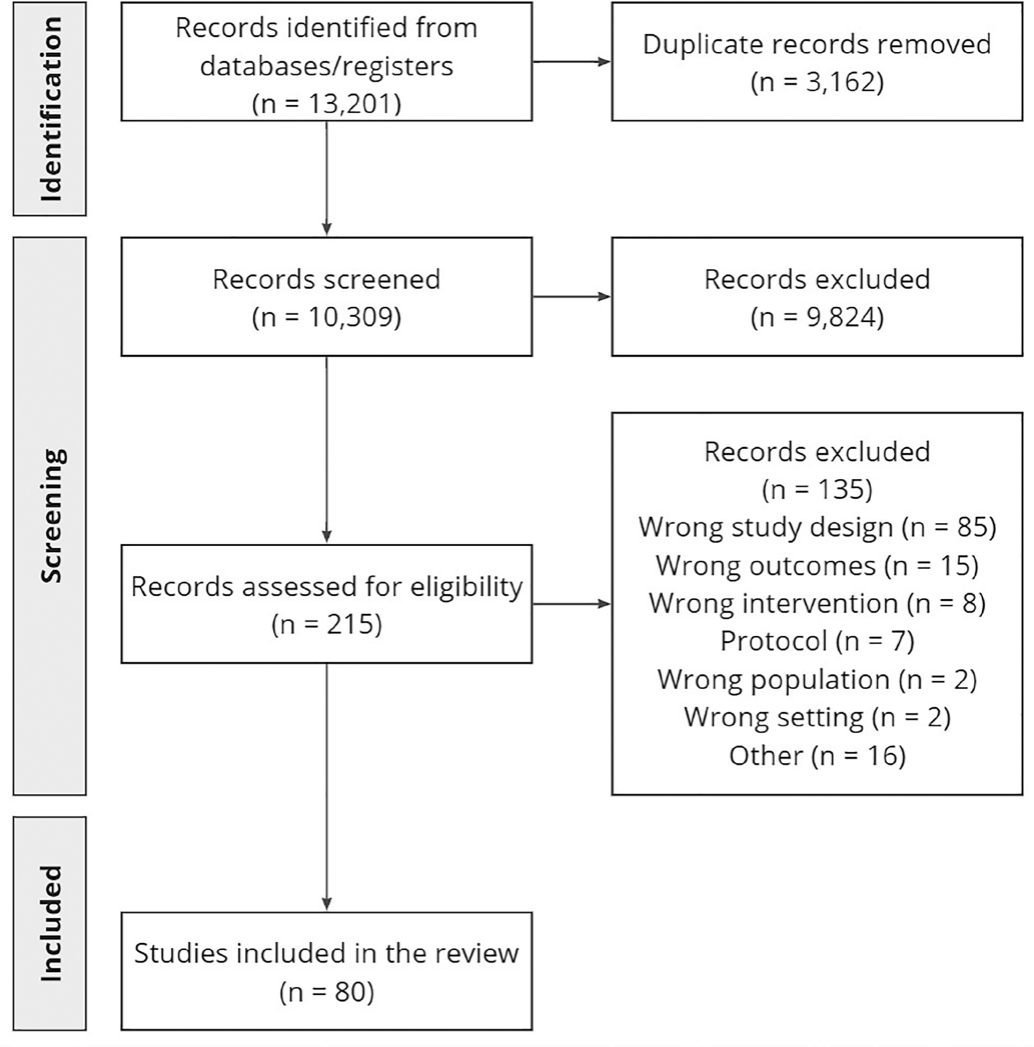
Preferred Reporting Items for Systematic Reviews and Meta-Analyses (PRISMA) flow diagram for the scoping review. PRISMA flow diagram illustrating the study selection process for the scoping review conducted to inform the development of best practice guidance for C-reactive protein point-of-care testing in the management of lower respiratory tract infections in primary care.

A total of 80 studies were included in the scoping review (figure 1), 65% of which focused on CRP as the POCT for improving antibiotic prescribing for RTIs in primary care. Procalcitonin was also considered, though it was used less frequently due to longer turnaround times and higher costs, making it less practical for routine use in primary care.^27^

### Delphi round 1

The Delphi process began with 49 statements, derived from the research team’s discussions and literature. In the first round, 19 of the 25 invited panel members completed the questionnaire, with 31 statements reaching consensus. Of these, 16 were accepted as guidance statements for using CRP POCTs in the management of LRTIs and 15 were accepted but slightly revised based on expert feedback. 18 statements did not reach consensus; nine were removed and nine were revised.

### Delphi round 2

In the second round, 24 statements (15 accepted but reworded and nine revised) were sent to all 19 members, and 16 experts completed the round. 17 statements reached consensus, while seven did not.

### Delphi round 3

The third round, completed by 12 of the 16 members, included the remaining seven statements, with consensus reached on four statements and three statements rejected.

### Accepted statements

The panel agreed that CRP POCT should be used in general practice to guide antibiotic prescribing decisions for LRTIs, particularly when prescribers are uncertain after a thorough assessment of the patient’s history, risk profile and clinical presentation. Consensus was reached on specific CRP cut-off values and their clinical implications, highlighting the role of CRP POCT in reducing diagnostic uncertainty while ensuring appropriate antibiotic use. The importance of integrating validated clinical decision rules (eg, STARWAVe, CRB-65) into patient assessments where appropriate was also emphasised to support evidence-based decision-making.

The panel acknowledged the ideal test characteristics, such as high sensitivity and specificity, as set out in statements regarding CRP POCT performance. However, these reflect optimal conditions rather than the actual diagnostic performance of CRP POCT in clinical practice, where trade-offs between sensitivity and specificity exist. While the expectation is for CRP POCTs to provide accurate and timely results, their interpretation should always be contextualised within a comprehensive clinical assessment, particularly given the potential for confounding factors (eg, inflammatory conditions that elevate CRP levels). This emphasised the importance of clear, structured guidelines to ensure the appropriate use of CRP POCT, in alignment with the test’s capabilities and the clinical context.

Further consensus was reached on the operational aspects of CRP POCT devices, emphasising the need for a streamlined testing process, minimal sample volume and efficient integration into electronic health records to facilitate ease of use. To ensure effective implementation, the panel highlighted the necessity of comprehensive training for healthcare professionals—not only on test operation and interpretation but also in communication skills to effectively engage patients in discussions about antibiotic stewardship and self-care within the context of LRTI management. The inclusion of CRP POCT alongside rapid viral testing during epidemics was also supported, contingent on test reliability and clinical relevance. Finally, statements were accepted on the need for government reimbursement of CRP POCT devices, consumables and provider services, recognising that financial considerations may influence adoption in routine practice.

### Rejected statements

The three rejected statements in the third round focused on the role of community pharmacists in performing CRP POCT, interpreting results and prescribing antibiotics. Concerns about fragmentation of care and the potential for undermining antibiotic stewardship efforts led to the rejection of these statements. These included: (1) performing CRP POCT in community pharmacies with appropriate training, (2) interpreting CRP POCT results by community pharmacists for LRTI diagnosis and (3) allowing community pharmacists to prescribe antibiotics based on CRP POCT results (full statements provided in online supplemental file 4).

### Additional information

**Figure 2:**
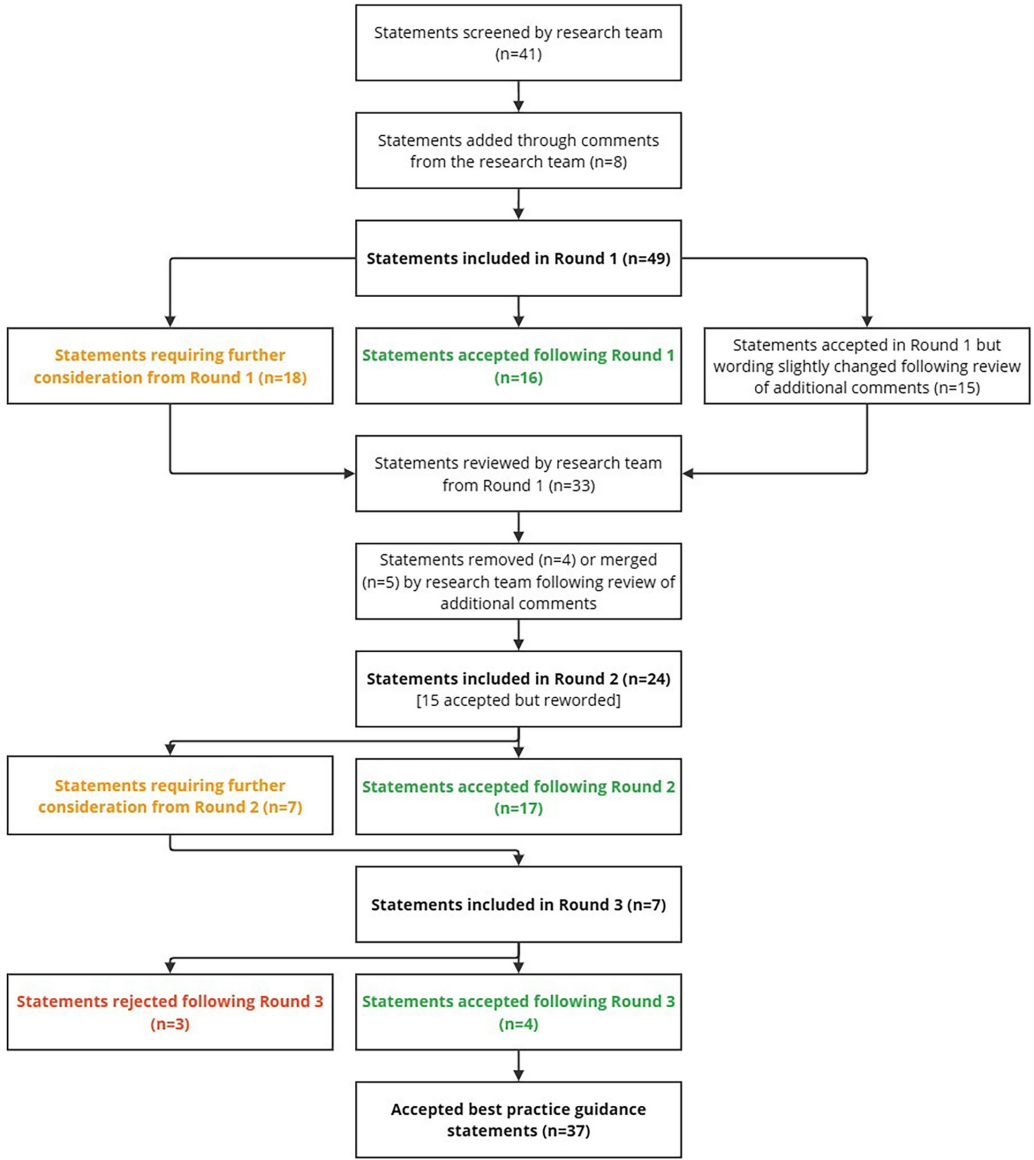
Flow diagram outlining the three-round Delphi process used to develop consensus-based best practice guidance statements for the use of C-reactive protein point-of-care tests in the management of lower respiratory tract infections in primary care.

Table 1 presents participant demographics, professional background and their experience with CRP POCT, providing context for the Delphi process, which is further detailed in figure 2. Raw data from all rounds are provided in onlinesupplemental files 7^9^, with comments from rounds 1 and 2 in onlinesupplemental files 5  6. The final set of criteria includes 37 guidance statements, categorised as follows: the use of the POCT (n=13); the detection of bacterial LRTIs using the POCT and the provision of antibiotics (n=7); communication strategies to increase antibiotic stewardship (n=4); features and performance of the point-of-care device (n=9); and user operation of the POCT (n=4). These are presented in table 2.

**Table 1:** Participant demographics and professional background

	Round 1	Round 2	Round 3
**Professional background**	**N (%)**	**N (%)**	**N (%)**
General practitioner	7 (36.8)	6 (32.5)	6 (50.0)
Hospital antimicrobial pharmacist	4 (21.1)	4 (25.0)	2 (16.7)
Community/academic pharmacist	6 (31.6)	5 (31.3)	4 (33.3)
Respiratory physician	1 (5.3)	1 (6.3)	0 (0)
Hospital pharmacist	1 (5.3)	0 (0)	0 (0)
**Duration in field of work**	**Mean**	**Mean**	**Mean**
Years	14.6	15	14.3
**Country of residence**	**N (%)**	**N (%)**	**N (%)**
Ireland	19 (100)	16 (100)	12 (100)
**Gender**	**N (%)**	**N (%)**	**N (%)**
Female	13 (68.4)	10 (62.5)	7 (58.3)
**Used CRP POCT before**	**N (%**)	**N (%**)	**N (%**)
No	19 (100)	16 (100)	12 (100)

CRP, C reactive protein; POCT, point-of-care test.

**Table 2:** Accepted best practice guidance statements for C-reactive protein (CRP) point-of-care test (POCT) use in primary care management of lower respiratory tract infections (LRTIs) from a three-round Delphi study

#	Statement
**Section 1: use of the POCT (n=13**)	
1	A thorough clinical assessment of the patient’s history (eg, comorbidities, previous admissions), risk profile and acute clinical situation should precede CRP POCT.
2	Validated, evidence-based clinical decision rules (eg, STARWAVe and CRB-65) should be incorporated into the clinical assessment of patients suspected of having a LRTI.
3	CRP POCT should be used for patients suspected of having a LRTI only when the prescriber is uncertain about prescribing antibiotics for LRTIs following their clinical assessment.
4	CRP POCT should aid appropriate prescribing of antibiotics for LRTIs by reducing diagnostic uncertainty as part of the clinical assessment.
5	Consent, either written or implied, should be obtained from the patient or the patient’s parent or legal guardian in the case of children below the age of 16 for their blood sample to be taken for CRP POCT. Additionally, where appropriate, children should provide their assent.
6	CRP POCT results should be interpreted with caution in patients with existing conditions that can elevate CRP values (eg, arthritis, gout, IBD) and in those receiving immunotherapy.
7	CRP POCT should be performed within general practice by general practitioners (GPs), guided by clinical judgement and current best practice guidance.
8	With appropriate training for the clinical assessment of patients suspected of having an LRTI, healthcare professionals in general practice, other than GPs themselves, should be able to obtain and interpret CRP POCT results with GP oversight.
9	With appropriate training for treating patients suspected of having an LRTI, healthcare professionals in general practice other than GPs themselves should be able to act on CRP POCT results with GP oversight.
10	CRP POCT should be used in conjunction with a rapid viral test in the presence of cough and fever during an active pandemic or epidemic. This is contingent on the tests demonstrating appropriate sensitivity, specificity and suitability for the context and population.
11	The cost of the CRP POCT device should be reimbursed by governments.
12	The cost of the CRP POCT consumables should be reimbursed by governments.
13	CRP POCT providers (ie, GPs and/or CPs) should be reimbursed by governments.
**Section 2: the detection of bacterial LRTIs using the POCT and the provision of antibiotics (n=7**)	
14	CRP values >20 mg/L may indicate the possibility of a bacterial LRTI, though clinical judgement should be used as CRP levels can also be elevated in viral infections (although CRP elevations due to viral infections typically remain below 20 mg/L).
15	CRP values <20 mg/L may indicate a self-limiting infection (bacterial/viral) for which antibiotics should not be prescribed.*
16	In cases where a child is presented early in the progression of symptoms (ie, in the first 24 hours), CRP results should be interpreted carefully, with attention to the clinical context and severity of illness. In this instance, a CRP value <5 mg/L may indicate a self-limiting infection (bacterial or viral) for which antibiotics should not be prescribed.
17	CRP values >100 mg/L in adults indicate the presence of a severe infection for which antibiotics should be prescribed following national/international antibiotic prescribing guidance and hospital referral considered.†
18	CRP values >75 mg/L in children indicate the presence of a severe infection for which antibiotics should be prescribed. following national/international antibiotic prescribing guidance and hospital referral considered.†
19	For CRP values in the range 20–100 mg/L in low-risk patients (ie, not at a higher risk of deterioration due to existing conditions or severe symptoms), prescribing of antibiotics should be avoided or delayed following national/international antibiotic prescribing guidance.*
20	For CRP values in the range 20–100 mg/L in patients with a higher risk of deterioration (ie, due to existing conditions or severe symptoms), antibiotic prescribing following national/international antibiotic prescribing guidance should be considered.†
**Section 3: communication strategies to increase antibiotic stewardship (n=4**)	
21	Patients should be informed about antibiotic resistance, the role (or lack thereof) of antibiotics in treating LRTIs and antibiotic stewardship.
22	CRP POCT results can be used to support patient-healthcare professional communication, especially when explaining whether antibiotics are required for a LRTI.
23	For CRP POCT conducted in community pharmacies, patients should be provided with a detailed description of the assessment undertaken and the results obtained so they can provide these to their GP if necessary.
24	CRP POCT should be used together with enhanced communication skills training.
**Section 4: features and performance of the point-of-care device (n=9**)	
25	CRP POCT results should have a high sensitivity.
26	CRP POCT results should have a high specificity.
27	CRP POCT results should have a high positive predictive value.
28	CRP POCT results should have a high negative predictive value.
29	Detection of a possible bacterial LRTI directly from a patient’s blood sample using CRP POCT should be completed in a one-step process.
30	CRP POCT should require a small sample volume of blood.
31	The space required for the CRP POCT device and operation should be minimal.
32	Results should be stored on the CRP POCT device or the PMR, with consideration for ease of use and integration into clinical workflow, while ensuring patient privacy and data security.
33	CRP POCT results should be automatically transferred from the device to patients’ records where feasible and efficient, leveraging fully integrated electronic healthcare records or easily scannable formats, to streamline documentation and ensure accurate record-keeping.
**Section 5: user operation of the POCT (n=4**)	
34	CRP POCT providers (eg, GPs, CPs and advanced nurse practitioners) should undergo training to use the POCT device and to interpret CRP results, combined with enhanced communication skills training to effectively convey findings and implications to patients.
35	The time required by staff to run the CRP POCT should be minimal.
36	Staff operation of the CRP POCT should follow the steps outlined by the manufacturer of the POCT device being used.
37	Maintenance, calibration and quality control will be required for the CRP POCT device as per manufacturer recommendations, with support and oversight provided by device supplier.

* Though clinical judgement and patient-specific factors should be considered, with clear communication of self-care and re-referral advice to the patient.

† Alongside thorough clinical assessment, with attention to the clinical context, severity of illness and potential non-bacterial causes of elevated CRP levels.

CP, community pharmacist; IBD, inflammatory bowel disease; PMR, patient medication record.

## Discussion

### Summary

Using the Delphi consensus method, this study developed 37 best practice guidance statements for using CRP POCT in the management of LRTIs in primary care. An expert panel facilitated the development of the guidance statements through their level of agreement, with revisions informed by panel feedback. Over three Delphi rounds, consensus was reached on key guidance statements, which provide a structured approach to incorporating CRP POCT in primary care. Their implementation could enhance antibiotic prescribing practices and support antibiotic stewardship efforts.

### Strengths and limitations

This study benefits from several strengths, including a rigorous scoping review that provided a solid foundation for the initial guidance statements. This evidence-informed approach strengthened the validity of the Delphi process, grounding the statements for CRP POCT use in the management of RTIs in primary care in robust, peer-reviewed research. The Delphi method, with its structured approach to consensus-building, ensured the iterative refinement of statements via feedback from healthcare professionals involved in the management of RTIs. Predefined criteria for acceptance, rejection or revision were used, following similar approaches used in other studies.^44^,^46^ Furthermore, panel members were given the opportunity to reflect on their individual ratings in round one and round two in conjunction with the group responses, which facilitated a thorough consideration of statements where consensus was not initially achieved.^39^

However, some limitations remain. As with all Delphi studies, reproducibility can be limited due to the reliance on the specific experts selected for the panel. Potential selection bias could arise from the expert panel being selected primarily through the researchers’ networks, which may have influenced the perspectives represented. Additionally, although response rates across Delphi rounds were consistent with the recommended range of 5–25 participants for optimal consensus,^44^,^50^ attrition from 19 participants in the first round to 12 in the final round could have affected the reliability of the final consensus. While the 12 remaining participants still represented a mix of healthcare professionals to provide diverse opinions, recent empirical work suggests that larger sample sizes may be required to achieve greater replicability of Delphi findings.^54^ Manyara *et al* recommend a minimum of 20–30 participants per stakeholder group to support moderate replicability in Delphi surveys, noting that smaller sample sizes (while common in the literature) may limit the stability of the results.^54^ Our smaller panel reflects pragmatic challenges of recruiting busy clinicians in an emerging area of practice, but we acknowledge this as a methodological limitation.

It is also important to acknowledge that none of the panellists had prior experience using CRP POCT in practice, as the technology is not yet widely available in Irish primary care (table 1). This likely influenced the perspectives captured and may have resulted in different results than if experienced users had participated. Future research should seek to validate and refine these guidance statements in real-world clinical settings, including input from healthcare professionals with hands-on experience using CRP POCT. Furthermore, the healthcare contexts in which the study participants were working—exclusively within Ireland—may limit the external validity of the findings to other settings, particularly those outside of Ireland or in healthcare systems with different resources and infrastructure.

### Comparison with existing literature

Previous studies using POCTs in the management of various conditions have highlighted important diagnostic and implementation considerations, providing valuable insights that contextualise our findings. Tong-Minh *et al* evaluated a POCT combining CRP and myxovirus resistance protein A to differentiate bacterial from viral RTIs in the emergency department setting, emphasising diagnostic accuracy through sensitivity, specificity and predictive values, as critical performance indicators.^55^ Their study highlights the importance of reliability, a core criterion in our guidance statements for CRP POCTs for LRTIs in primary care. Additionally, their single-use finger-prick test delivered results within 10 min,^55^ reinforcing the need for rapid turnaround times, a key factor identified in our study.

Weir *et al* used a Delphi consensus approach to establish POCT criteria for urinary tract infections to support appropriate antibiotics use, demonstrating the effectiveness of this method in generating structured, expert-driven guidance.^44^ Their study parallels our approach, underscoring the value of systematically developing POCT guidance statements tailored to clinical needs. Similarly, Hsieh *et al* identified key characteristics for POCTs to detect sexually transmitted infections, including ease of use, rapid turnaround times (preferably within 5 min), user-friendliness and minimal disruption to clinical workflows.^56^ These findings align with the present study, where user-friendliness, efficiency and operational feasibility were highlighted as essential for CRP POCT integration into primary care.

De Vos *et al* explored the implementation of rapid *Neisseria gonorrhoeae* POCTs in South African primary care, emphasising the importance of clear communication, discretion, speed and accuracy in fostering patient and healthcare professional acceptance.^57^ Furthermore, the need for comprehensive training and supportive materials for healthcare professionals, to ensure successful adoption, was identified.^57^ These considerations were reflected in our guidance statements, which stress the role of training, communication and patient education in supporting antibiotic stewardship. Figueira *et al* examined POCTs for HIV, hepatitis C and hepatitis B infections in community pharmacies, identifying accessibility, speed and confidentiality as key facilitators, but also highlighting barriers such as staffing constraints, logistical challenges and inadequate funding.^58^ These barriers align with our guidance statements, which emphasise the importance of funding (statements 11–13).

Overall, our study builds on this body of literature by developing a consensus-based set of guidance statements that address diagnostic accuracy, workflow integration, patient communication and user training. Additionally, our findings provide practical recommendations on acceptable CRP thresholds for decision-making, reinforcing the broader applicability of POCTs in primary care settings.

### Implications for policy and practice

While there is considerable interest in the use of POCTs to aid in diagnosing infections in primary care, their successful integration depends on delivering accurate, reliable, timely and cost-effective results, as highlighted by our findings. The literature suggests that POCTs are most effective when used within clear, evidence-based guidelines that outline their appropriate indications and interpretations. The implications of this Delphi study are significant for healthcare professionals and policymakers alike. For healthcare professionals, our validated guidance statements provide a practical framework for utilising CRP POCTs in diagnosing and managing LRTIs. This approach can support more informed antibiotic prescribing, aiming to reduce unnecessary antibiotic use and ultimately improve patient care for those presenting with LRTI symptoms. Promoting the appropriate use of antibiotics remains a critical patient safety and public health priority globally.

Several international reports and expert commissions have called for the integration of POCTs in antimicrobial stewardship efforts. The 2020 Wellcome Trust AMR report and the UK’s ‘Review on Antimicrobial Resistance’ emphasised the importance of POCTs that distinguish between viral and bacterial infections as a key strategy in combating AMR.^59 60^ Furthermore, the WHO has advocated for better diagnostic testing to support AMR efforts, reinforcing the role of POCTs in reducing the unnecessary use of antibiotics.^61^ A potential tension exists between the guidance statements developed in this study and international recommendations such as those made in O’Neill’s Review on Antimicrobial Resistance, which called for high-income countries to make it ‘mandatory that by 2020 the prescription of antibiotics will need to be informed by data and testing technology wherever it is available.’^60^ This recommendation reflects a push for widespread implementation of diagnostic testing to guide antibiotic use. In contrast, our Delphi panel reached consensus that CRP POCT should be used specifically when the prescriber is uncertain about whether to prescribe an antibiotic for a suspected LRTI following clinical assessment, reflecting a more selective, targeted approach to POCT use.

The implementation of CRP POCTs, guided by evidence-based recommendations, aligns with national antimicrobial stewardship initiatives, with a focus on integrating diagnostic tools like POCTs into clinical assessment pathways to help differentiate viral from bacterial infections.^62^ This integration could substantially reduce unnecessary antimicrobial prescribing and contribute to global AMR goals. However, enabling the widespread uptake of CRP POCTs in primary care will require significant investment, including funding for device procurement, training for healthcare professionals and the development of reimbursement models that support their use. Policymakers must consider these factors to ensure that CRP POCTs can be effectively adopted and integrated into primary care settings, as highlighted by our guidance statements.

### Future research

The guidance developed in this study aims to address a critical gap in primary care by standardising the use of CRP POCT, where a lack of clear protocols and reimbursement mechanisms has previously hindered its widespread implementation. This guidance highlights the need for comprehensive training for healthcare professionals, covering both the technical aspects of CRP POCT device operation and effective communication strategies for discussing results with patients. Successful integration of CRP POCT will rely on its ability to deliver accurate, reliable, timely and cost-effective results, alongside access to the necessary technology and robust support to facilitate its incorporation into healthcare workflows. This includes educating both healthcare professionals and patients about the purpose and implications of CRP testing.

Future research should focus on evaluating the real-world impact of these guidance statements, particularly in relation to prescribing behaviours, patient outcomes and healthcare costs. Pilot studies in general practice and community pharmacy settings are needed to assess the acceptability, feasibility and effectiveness of the proposed guidance in reducing unnecessary antibiotic use. Additionally, further exploration of patient perceptions and acceptance of CRP POCT, as well as its influence on patient-provider interactions, will be essential for optimising its use and ensuring seamless integration into routine clinical practice.

## Conclusion

The lack of guidance to support the use of CRP POCT in the management of LRTIs has been identified as one of the barriers to its widespread use. This study developed a set of consensus-based best practice guidance statements, informed by the Delphi technique, to support the appropriate use of CRP POCT in primary care. Consensus was reached on statements covering the test’s role in guiding antibiotic prescribing, its ability to detect bacterial LRTIs, communication strategies to enhance antibiotic stewardship, device features and performance and operational considerations for implementation. Given the complexity of antibiotic prescribing—shaped by diagnostic uncertainty, patient expectations, time constraints and external pressures—these best practice guidance statements provide a structured framework to support clinical decision-making. By ensuring that CRP POCT is used within a clear, evidence-based framework, alongside enhanced communication strategies, these statements could facilitate more rational antibiotic prescribing for patients with LRTI symptoms in primary care.

## Supplementary material

10.1136/bmjopen-2025-101438 online supplemental file 1

10.1136/bmjopen-2025-101438 online supplemental file 2

10.1136/bmjopen-2025-101438 online supplemental file 3

10.1136/bmjopen-2025-101438 online supplemental file 4

10.1136/bmjopen-2025-101438 online supplemental file 5

10.1136/bmjopen-2025-101438 online supplemental file 6

10.1136/bmjopen-2025-101438 online supplemental file 7

10.1136/bmjopen-2025-101438 online supplemental file 8

10.1136/bmjopen-2025-101438 online supplemental file 9

## Data Availability

All data relevant to the study are included in the article or uploaded as supplementary information.
