# Supplementary material for: Point-of-caRE DiagnostICs for respiraTOry tRact infectionS (PREDICTORS) study: developing guidance for using C-reactive protein point-of-care tests in the management of lower respiratory tract infections in primary care using a Delphi consensus technique
Source: BMJ Open. 2025 May 27;15(5):e101438. doi: 10.1136/bmjopen-2025-101438 (PMC12121597; doi:10.1136/bmjopen-2025-101438)
Supplement: online supplemental file 1 [file bmjopen-15-5-s001.docx]

# Supporting Information File 1: DELPHISTAR Checklist

Delphi studies in social and health sciences – recommendations for an interdisciplinary standardized reporting (DELPHISTAR)

| **Topic** | **Section** | **Item** | **Checklist Item** | **Location where item is reported** |
| --- | --- | --- | --- | --- |
| **I**  **Title and Abstract** |  | 1 | Identification as a Delphi study in the title | Page 1 |
|  |  | 2 | Identification as a Delphi study in the abstract | Page 2 |
|  |  | 3 | Structured abstract | Page 2 |
| **II**  **Context** | **Formal** | 4 | Information about the sources of funding | Page 18 |
|  |  | 5 | Information about the team of authors and/or researchers (e.g., discipline, institution) | Page 1 |
|  |  | 6 | Information about method consulting | Page 6 |
|  |  | 7 | Information about the project background | Page 5 |
|  |  | 8 | Information about the study protocol | Page 17 |
|  | **Content** | 9 | Justification of the chosen method (Delphi) to answer the research question | Page 6 |
|  |  | 10 | Aim of the Delphi study (e.g., consensus, forecasting) | Page 5 |
| **III**  **Method** | **Body & Integration of knowledge** | 11 | Identification and elucidation of relevant expertise, spheres of experience, and perspectives (e.g., theory, practice, affected groups, disciplines) | Page 7 |
|  |  | 12 | Handling of knowledge, expertise and perspectives which are missing or have been deliberately not integrated | Page 12 |
|  |  | 13 | Basic definition of expert^1^ | Page 7 |
|  | **Delphi variant and modifications** | 14 | Identification of the type of Delphi variant and potential modifications (e.g., classic Delphi, real-time Delphi, group Delphi) | Page 6 |
|  |  | 15 | Justification of the Delphi variant and modifications, including during the Delphi study, if applicable | Page 8 |
|  | **Sample of experts** | 16 | Selection criteria for the experts (per round, per expert group if applicable) | Page 7 |
|  |  | 17 | Identification of the experts | Page 7 |
|  |  | 18 | Information about recruiting and any subsequent recruiting of experts | Page 7 |
|  | **Survey** | 19 | Elucidation of the content development for the questionnaire^2^ | Page 6 |
|  |  | 20 | Description of the questionnaire (content and structure) | Page 8 |
|  | **Delphi rounds** | 21 | Number of Delphi rounds | Page 8 |
|  |  | 22 | Information about the aims of the individual Delphi rounds | Page 8 |
|  |  | 23 | Disclosure and justification of the criterion for discontinuation | Page 8 |
|  | **Feedback** | 24 | Information about what data was reported back per round | Page 8 |
|  |  | 25 | Information on how the results of the previous Delphi round were fed back to the experts surveyed (e.g., via frequencies, mean values, measures of dispersion, listing of comments) | Page 8 |
|  |  | 26 | Information on whether feedback was differentiated by specific groups (e.g., by field of expertise, institutional affiliation) | N/A |
|  |  | 27 | Information about how dissent and unclear results were handled | Page 8 |
|  | **Data analysis** | 28 | Disclosure of the quantitative and qualitative analytical strategy | Page 8 |
|  |  | 29 | Definition and measurement of consensus | Page 8 |
|  |  | 30 | Information on group-specific analysis or weighting of experts (e.g., theory vs. practice, discipline-specific analysis) | N/A |
| **IV**  **Results** | **Delphi process** | 31 | Illustration of the Delphi study (e.g., in a flow chart) | Page 11 |
|  |  | 32 | Information about special aspects during the Delphi study (e.g., deviations from the intended approach with justification) | N/A |
|  |  | 33 | Number of experts per round (both invited and participating) | Page 9 |
|  | **Results** | 34 | Presentation of the results for each Delphi round and the final results | Page 9 |
| **V Discussion** | **Quality of findings** | 35 | Highlighting the findings from the Delphi study | Page 12 |
|  |  | 36 | Validity of the results (e.g., transferability of the findings) | Page 12 |
|  |  | 37 | Reliability of the results (e.g., split half, inter-rater reliability) | Page 8 |
|  |  | 38 | Reflection on potential limitations (e.g., number of experts, response bias) | Page 12 |
