# Supplementary material for: Point-of-caRE DiagnostICs for respiraTOry tRact infectionS (PREDICTORS) study: developing guidance for using C-reactive protein point-of-care tests in the management of lower respiratory tract infections in primary care using a Delphi consensus technique
Source: BMJ Open. 2025 May 27;15(5):e101438. doi: 10.1136/bmjopen-2025-101438 (PMC12121597; doi:10.1136/bmjopen-2025-101438)
Supplement: online supplemental file 2 [file bmjopen-15-5-s002.docx]

# Supporting Information File 2: Round 1 Questionnaire

## Section 1: Using CRP POCT

| 1. **CRP POCT should be used for all patients suspected of having a lower respiratory tract infection (LRTI) regardless of age or demographics.**   Rationale: CRP POCT can help reduce inappropriate antibiotic prescribing (1-11). A CRP POCT can give more certainty about the presence/absence of a bacterial RTI and guide the decision to prescribe/avoid an antibiotic (1). | | | | |
| --- | --- | --- | --- | --- |
| 1: Strongly disagree | 2: Disagree | 3: Uncertain | 4: Agree | 5: Strongly agree |
| Comment: | | | | |

| 1. **CRP POCT should be used for patients suspected of having a LRTI only when the prescriber is uncertain about prescribing antibiotics for LRTIs.**   Rationale: CRP POCT can help reduce inappropriate antibiotic prescribing (1-11). A CRP POCT can give more certainty about the presence/absence of a bacterial LRTI and guide the decision to prescribe/avoid an antibiotic (1). | | | | |
| --- | --- | --- | --- | --- |
| 1: Strongly disagree | 2: Disagree | 3: Uncertain | 4: Agree | 5: Strongly agree |
| Comment: | | | | |

| 1. **CRP POCT should be used for patients suspected of having a LRTI when the prescriber is thinking about prescribing antibiotics for LRTIs.**   Rationale: CRP POCT can help reduce inappropriate antibiotic prescribing (1-11). A CRP POCT can give more certainty about the presence/absence of a bacterial LRTI and guide the decision to prescribe/avoid an antibiotic (1). | | | | |
| --- | --- | --- | --- | --- |
| 1: Strongly disagree | 2: Disagree | 3: Uncertain | 4: Agree | 5: Strongly agree |
| Comment: | | | | |

| 1. **CRP POCT results should be interpreted with caution in patients with existing conditions that can elevate CRP values (*e.g.,* arthritis, gout, inflammatory bowel disease) and in those receiving immunotherapy.**   Rationale: Autoimmune diseases (arthritis, inflammatory bowel disease), malignant neoplasms, gout, tissue injury and immunosuppressive therapy raise CRP values, independent of the presence of bacterial infection (3). | | | | |
| --- | --- | --- | --- | --- |
| 1: Strongly disagree | 2: Disagree | 3: Uncertain | 4: Agree | 5: Strongly agree |
| Comment: | | | | |

| 1. **CRP POCT should reduce diagnostic uncertainty as part of the assessment for LRTIs and aid prescribing decisions.**   Rationale: CRP is a sensitive and non-specific marker for inflammation which can be used to assess the severity of an inflammation and to predict if an infection can be expected to be self-limiting or severe (1). Uncertainty about the diagnosis of infection can lead to inappropriate antibiotic prescribing (12). CRP POCT helps to assess bacterial LRTI severity and to identify the patients who may benefit from antibiotics (1-4). | | | | |
| --- | --- | --- | --- | --- |
| 1: Strongly disagree | 2: Disagree | 3: Uncertain | 4: Agree | 5: Strongly agree |
| Comment: | | | | |

| 1. **CRP POCT results should be interpreted in conjunction with a thorough assessment of the patient’s history, risk profile, and acute clinical situation.**   Rationale: Patients' comorbidities and sources of vulnerability/risk should be considered in conjunction with CRP-based prescribing recommendations before deciding on treatment (1, 2). | | | | |
| --- | --- | --- | --- | --- |
| 1: Strongly disagree | 2: Disagree | 3: Uncertain | 4: Agree | 5: Strongly agree |
| Comment: | | | | |

| 1. **Clinical decision rules that are proven for use in your practice should be incorporated into the clinical assessment of patients suspected of having a LRTI.**   Rationale: Using clinical decision rules (for example, [FeverPAIN](https://bmjopen.bmj.com/content/3/10/e003943)) provides confirmation of a bacterial LRTI and its severity and helps to identify patients requiring further referral (13). | | | | |
| --- | --- | --- | --- | --- |
| 1: Strongly disagree | 2: Disagree | 3: Uncertain | 4: Agree | 5: Strongly agree |
| Comment: | | | | |

| 1. **CRP POCT should be performed within general practice.**   Rationale: Patients frequently attend general practice with symptoms of LRTIs; therefore, using a CRP POCT in this setting should help to ensure appropriate prescribing of antibiotics. | | | | |
| --- | --- | --- | --- | --- |
| 1: Strongly disagree | 2: Disagree | 3: Uncertain | 4: Agree | 5: Strongly agree |
| Comment: | | | | |

| 1. **Healthcare professionals in general practice, other than general practitioners (GPs) themselves (*i.e.,* advanced nurse practitioners, general practice pharmacists) should be able to obtain and interpret CRP POCT results.**   Rationale: Having other healthcare professionals involved in diagnosing and managing LRTIs should reduce the workload/pressure on GPs (13). | | | | |
| --- | --- | --- | --- | --- |
| 1: Strongly disagree | 2: Disagree | 3: Uncertain | 4: Agree | 5: Strongly agree |
| Comment: | | | | |

| 1. **Healthcare professionals in general practice, other than GPs themselves (*i.e.,* advanced nurse practitioners, general practice pharmacists) should be able to act upon CRP POCT results (*i.e*., prescribe antibiotic therapy when indicated following national/international antibiotic prescribing guidance or provide self-care advice to patients).**   Rationale: Having other healthcare professionals involved in diagnosing and managing LRTIs should reduce the workload/pressure on GPs (13). | | | | |
| --- | --- | --- | --- | --- |
| 1: Strongly disagree | 2: Disagree | 3: Uncertain | 4: Agree | 5: Strongly agree |
| Comment: | | | | |

| 1. **The decision whether to perform CRP POCT and whether to prescribe antibiotics should remain at the discretion of the GP.**   Rationale: If CRP POCT becomes mandatory to reduce over-prescribing of antibiotics, GPs may be concerned over a perceived lack of confidence in their clinical assessment (3). | | | | |
| --- | --- | --- | --- | --- |
| 1: Strongly disagree | 2: Disagree | 3: Uncertain | 4: Agree | 5: Strongly agree |
| Comment: | | | | |

| 1. **CRP POCT should be performed within community pharmacies.**   Rationale: Community pharmacists are accessible in a timely manner and regularly provide self-care advice for minor ailments including LRTIs. | | | | |
| --- | --- | --- | --- | --- |
| 1: Strongly disagree | 2: Disagree | 3: Uncertain | 4: Agree | 5: Strongly agree |
| Comment: | | | | |

| 1. **Appropriately trained community pharmacists should be able to obtain and interpret CRP POCT results.**   Rationale: Community pharmacists are accessible in a timely manner. Enabling them to use CRP POCT in the management of LRTIs should improve patient experience and reduce pressure on general practice . | | | | |
| --- | --- | --- | --- | --- |
| 1: Strongly disagree | 2: Disagree | 3: Uncertain | 4: Agree | 5: Strongly agree |
| Comment: | | | | |

| 1. **Appropriately trained community pharmacists should be able to act upon CRP POCT results (*i.e.,* prescribe antibiotic therapy when indicated following national/international antibiotic prescribing guidance or provide self-care advice to patients).**   Rationale: Community pharmacists are accessible in a timely manner. Enabling them to use CRP POCT in the management of LRTIs should improve patient experience and reduce pressure on general practice . | | | | |
| --- | --- | --- | --- | --- |
| 1: Strongly disagree | 2: Disagree | 3: Uncertain | 4: Agree | 5: Strongly agree |
| Comment: | | | | |

| 1. **Group A strep testing for patients presenting with a sore throat is more suitable for use within community pharmacies than CRP POCT for LRTIs.**   Rationale: While both strep A and CRP POCT have their merits in guiding treatment decisions for LRTIs, strep A testing may be more relevant in community pharmacies. As the majority of sore throats are caused by a virus, patients can be offered self-care in the pharmacy (14). | | | | |
| --- | --- | --- | --- | --- |
| 1: Strongly disagree | 2: Disagree | 3: Uncertain | 4: Agree | 5: Strongly agree |
| Comment: | | | | |

| 1. **The decision whether to perform CRP POCT should remain at the discretion of the community pharmacist.**   Rationale: If CRP POCT becomes mandatory to reduce over-prescribing of antibiotics, community pharmacists may be concerned over a perceived lack of confidence in their clinical assessment (3). | | | | |
| --- | --- | --- | --- | --- |
| 1: Strongly disagree | 2: Disagree | 3: Uncertain | 4: Agree | 5: Strongly agree |
| Comment: | | | | |

| 1. **Following clinical assessment of patients presenting with symptoms of a LRTI, GPs should refer patients to community pharmacy for CRP POCT with a prescription on-hold for antibiotics.**   Rationale: The community pharmacist can perform CRP POCT on behalf of the GP following the GP’s clinical assessment of the patient and dispense the prescription if the CRP POCT indicates a bacterial LRTI. | | | | |
| --- | --- | --- | --- | --- |
| 1: Strongly disagree | 2: Disagree | 3: Uncertain | 4: Agree | 5: Strongly agree |
| Comment: | | | | |

| 1. **Consent will be provided by the patient or the patient’s parent or legal guardian in the case of children below the age of 16 for their blood sample to be taken for CRP POCT.**   Rationale: As with all diagnostic tests, consent will be required for CRP POCT. | | | | |
| --- | --- | --- | --- | --- |
| 1: Strongly disagree | 2: Disagree | 3: Uncertain | 4: Agree | 5: Strongly agree |
| Comment: | | | | |

| 1. **CRP POCT should be used in conjunction with a rapid viral test (e.g., SARS-Cov-2/Influenza A&B/RSV Adenovirus antigen combo rapid test) in the presence of cough and fever during an active pandemic or epidemic (such as COVID-19 or Influenza A&B).**   Rationale: In this instance, a rapid viral test could be used to aid interpretation of CRP POCT results (1). For patients that have tested positive for COVID-19, CRP POCT can be useful for disease prognosis (1, 3); in these patients, raised CRP >40 mg/L is indicative of severe infections, indicating the need for close follow-up or hospitalisation (15). | | | | |
| --- | --- | --- | --- | --- |
| 1: Strongly disagree | 2: Disagree | 3: Uncertain | 4: Agree | 5: Strongly agree |
| Comment: | | | | |

| 1. **The cost of the CRP POCT device should be reimbursed by Governments.**   Rationale: Uptake of new technologies in healthcare is often drive by underlying financial structures. Reimbursement can drive local access and use of CRP POCT (3). | | | | |
| --- | --- | --- | --- | --- |
| 1: Strongly disagree | 2: Disagree | 3: Uncertain | 4: Agree | 5: Strongly agree |
| Comment: | | | | |

| 1. **The cost of the CRP POCT consumables should be reimbursed by Governments.**   Rationale: In the Netherlands, the consumables to operate CRP POCT is reimbursed within their national health services (3). | | | | |
| --- | --- | --- | --- | --- |
| 1: Strongly disagree | 2: Disagree | 3: Uncertain | 4: Agree | 5: Strongly agree |
| Comment: | | | | |

| 1. **CRP POCT providers (i.e., GPs and/or community pharmacists) should be reimbursed by Governments.**   Rationale: A fee-for-service reimbursement mechanism should spur broader CRP POCT implementation and ultimately improve patient access (3). | | | | |
| --- | --- | --- | --- | --- |
| 1: Strongly disagree | 2: Disagree | 3: Uncertain | 4: Agree | 5: Strongly agree |
| Comment: | | | | |

## Section 2: The detection of bacterial LRTIs using CRP POCT and the provision of antibiotics

| 1. **CRP POCT results can inform the need for antibiotic therapy in LRTIs.**   Rationale: CRP POCT should aid appropriate prescribing of antibiotics (1-11). A high CRP value (>100 mg/L) can indicate a severe bacterial infection. Antibiotic treatment can usually be avoided when the CRP value is low (<20 mg/L) (1-3). The interpretation of a CRP value should be made in addition to clinical assessment based on presenting signs and symptoms. | | | | |
| --- | --- | --- | --- | --- |
| 1: Strongly disagree | 2: Disagree | 3: Uncertain | 4: Agree | 5: Strongly agree |
| Comment: | | | | |

| 1. **A CRP value >20 mg/L indicates the possibility of a bacterial LRTI.**   Rationale: In patients without infection or morbidities that contribute to elevated CRP, serum CRP values are below 5 mg/L (16). Raised serum CRP values often occur in bacterial infections; however, typically only minor elevations are observed in viral infections (5). | | | | |
| --- | --- | --- | --- | --- |
| 1: Strongly disagree | 2: Disagree | 3: Uncertain | 4: Agree | 5: Strongly agree |
| Comment: | | | | |

| 1. **A CRP value <20 mg/L indicates a self-limiting infection (bacterial or viral) for which antibiotics should not be prescribed.**   Rationale: Self-limiting infections (viral or bacterial) are those that tend to resolve themselves without further treatment and represent the majority of LRTIs seen in primary care (1). Antibiotics should be withheld when the clinical assessment rules out a severe infection and CRP value is <20 mg/L (1-3, 11). Instead self-care advice can be given and patients advised to re-consult if symptoms worsen or persist (1, 2). | | | | |
| --- | --- | --- | --- | --- |
| 1: Strongly disagree | 2: Disagree | 3: Uncertain | 4: Agree | 5: Strongly agree |
| Comment: | | | | |

| 1. **CRP POCT should have a lower threshold for withholding antibiotics in children (<5 mg/L or <10 mg/L) than adults (<20 mg/L).**   Rationale: Parents of unwell children typically present early with their child in primary care. In these cases, CRP POCT results should be interpreted carefully (2). Various CRP thresholds for withholding antibiotics in children have been reported (<20 mg/L (2, 17), <10 mg/L (17, 18), or <5 mg/L (19)). | | | | |
| --- | --- | --- | --- | --- |
| 1: Strongly disagree | 2: Disagree | 3: Uncertain | 4: Agree | 5: Strongly agree |
| Comment: | | | | |

| 1. **CRP values >100 mg/L in adults indicates the presence of a severe infection for which antibiotics should be prescribed following national/international antibiotic prescribing guidance and hospital referral considered alongside thorough clinical assessment.**   Rationale: It is strongly recommended to start treatment with antibiotics due to a high risk of a severe bacterial infection at CRP value >100 mg/L (1-3) (pneumonia likely if one of the following are present: new focal chest signs, dyspnoea, tachypnoea, pulse rate >100, fever >4 days (11). Consider hospital referral alongside thorough clinical assessment if CRP value >100 mg/L (1). | | | | |
| --- | --- | --- | --- | --- |
| 1: Strongly disagree | 2: Disagree | 3: Uncertain | 4: Agree | 5: Strongly agree |
| Comment: | | | | |

| 1. **CRP values >75 mg/L in children indicates the presence of a severe infection for which antibiotics should be prescribed following national/international antibiotic prescribing guidance and hospital referral considered alongside thorough clinical assessment.**   Rationale: It is strongly recommended to start treatment with antibiotics due to a high risk of a non-self-limiting infection or refer to hospital (2, 19). Consider urgent hospital referral alongside thorough clinical assessment if CRP value >100 mg/L (2). | | | | |
| --- | --- | --- | --- | --- |
| 1: Strongly disagree | 2: Disagree | 3: Uncertain | 4: Agree | 5: Strongly agree |
| Comment: | | | | |

| 1. **For CRP values between 20-100 mg/L in low-risk patients (i.e., not at a higher risk of deterioration due to existing conditions or severe symptoms), prescribing of antibiotics should be avoided or delayed following national/international antibiotic prescribing guidance.**   Rationale: For CRP values between 20-100 mg/L, the clinical assessment of patients presenting with a LRTI remains the primary decision-driver in whether to prescribe an antibiotic (1, 2). In most cases, antibiotics are not needed. Delayed prescribing can be considered if illness severity did not require immediate antibiotics and CRP value is between 20-100 mg/L (1-5). | | | | |
| --- | --- | --- | --- | --- |
| 1: Strongly disagree | 2: Disagree | 3: Uncertain | 4: Agree | 5: Strongly agree |
| Comment: | | | | |

| 1. **For CRP values between 20-100 mg/L in patients with a higher risk of deterioration (i.e., due to existing conditions or severe symptoms), antibiotic prescribing following national/international antibiotic prescribing guidance should be considered.**   Rationale: For CRP values between 20-100 mg/L, prescribing of antibiotics should be considered in patients with relevant comorbidities, such as chronic obstructive pulmonary disease (exhibiting exacerbation with obvious increased purulence of sputum), diabetes and in vulnerable older patients (1). | | | | |
| --- | --- | --- | --- | --- |
| 1: Strongly disagree | 2: Disagree | 3: Uncertain | 4: Agree | 5: Strongly agree |
| Comment: | | | | |

## Section 3: Communication strategies to increase antibiotic stewardship

| 1. **Patients should be informed about antibiotic resistance, the role (or lack thereof) of antibiotics in treating LRTIs, and antibiotic stewardship.**   Rationale: The consultation for LRTI assessment is typically a moment when symptoms are acute, making the messaging around antibiotic usage relevant and timely (2). In this clinical encounter, healthcare professionals should inform patients about the appropriate use of antibiotics (20). | | | | |
| --- | --- | --- | --- | --- |
| 1: Strongly disagree | 2: Disagree | 3: Uncertain | 4: Agree | 5: Strongly agree |
| Comment: | | | | |

| 1. **CRP POCT results can be used to support patient-healthcare professional communication, especially when explaining whether antibiotics are required for a LRTI.**   Rationale: CRP test result and a well-informed prescribing decision should help reduce antibiotic over-consumption, but patients need to be informed about the POCT, outcomes based on test results, and its implication for antibiotic stewardship (1-3). | | | | |
| --- | --- | --- | --- | --- |
| 1: Strongly disagree | 2: Disagree | 3: Uncertain | 4: Agree | 5: Strongly agree |
| Comment: | | | | |

| 1. **For CRP POCT conducted in general practice, outcomes of the test should be provided to community pharmacists.**   Rationale: Depending on clinical assessment and CRP POCT results, GP communication with community pharmacists will help identify patients who require self-care and provide clarity on the necessity of prescriptions for antibiotics in the treatment of LRTIs. | | | | |
| --- | --- | --- | --- | --- |
| 1: Strongly disagree | 2: Disagree | 3: Uncertain | 4: Agree | 5: Strongly agree |
| Comment: | | | | |

| 1. **For CRP POCT conducted in community pharmacies, outcomes of the test should be provided to GPs.**   Rationale: Depending on clinical assessment and CRP POCT results, patients should either be provided advice on self-care in community pharmacies or referred to general practice for antibiotic prescriptions. CRP POCT results should be sent to the GP for transparency. | | | | |
| --- | --- | --- | --- | --- |
| 1: Strongly disagree | 2: Disagree | 3: Uncertain | 4: Agree | 5: Strongly agree |
| Comment: | | | | |

| 1. **CRP POCT should be used together with enhanced communication skills training.**   Rationale: While the use of CRP POCT reduces antibiotic prescribing, combining it with communication skills training (focused on antibiotic stewardship, CRP POCT, patient-centred consultations and shared decision-making) can significantly increase this impact (1-3, 21, 22). | | | | |
| --- | --- | --- | --- | --- |
| 1: Strongly disagree | 2: Disagree | 3: Uncertain | 4: Agree | 5: Strongly agree |
| Comment: | | | | |

## Section 4: Features and performance of the CRP POCT device

| 1. **CRP POCT results should have a high sensitivity.**   Rationale: High sensitivity means there is a high probability of correctly detecting the presence of a bacterial LRTI. | | | | |
| --- | --- | --- | --- | --- |
| 1: Strongly disagree | 2: Disagree | 3: Uncertain | 4: Agree | 5: Strongly agree |
| Comment: | | | | |

| 1. **CRP POCT results should have a high specificity.**   Rationale: High specificity means there is a high probability of accurately detecting the absence of a bacterial LRTI. | | | | |
| --- | --- | --- | --- | --- |
| 1: Strongly disagree | 2: Disagree | 3: Uncertain | 4: Agree | 5: Strongly agree |
| Comment: | | | | |

| 1. **CRP POCT results should have a high positive predictive value.**   Rationale: Positive predictive value: the likelihood that if a test indicates a condition is present (positive result), the person actually has that condition. High positive predictive value refers to the probability that those samples which indicate the presence of a bacterial LRTI based on CRP value with the POCT truly have a bacterial LRTI. | | | | |
| --- | --- | --- | --- | --- |
| 1: Strongly disagree | 2: Disagree | 3: Uncertain | 4: Agree | 5: Strongly agree |
| Comment: | | | | |

| 1. **CRP POCT results should have a high negative predictive value.**   Rationale: Negative predictive value: the likelihood that if a test indicates a condition is not present (negative result), the person truly does not have that condition. High negative predictive value refers to the probability that those samples which do not indicate the presence of a bacterial LRTI based on CRP value with the POCT truly do not have a bacterial LRTI. | | | | |
| --- | --- | --- | --- | --- |
| 1: Strongly disagree | 2: Disagree | 3: Uncertain | 4: Agree | 5: Strongly agree |
| Comment: | | | | |

| 1. **Detection of a possible bacterial LRTI directly from a patient’s blood sample using CRP POCT should be completed in a one-step process.**   Rationale: Once a sample has been loaded onto the device, no further input is required from the healthcare professional. | | | | |
| --- | --- | --- | --- | --- |
| 1: Strongly disagree | 2: Disagree | 3: Uncertain | 4: Agree | 5: Strongly agree |
| Comment: | | | | |

| 1. **CRP POCT should require a small sample volume of blood.**   Rationale: CRP value can be obtained from a finger-prick blood sample using approximately 20 microlitres of blood. | | | | |
| --- | --- | --- | --- | --- |
| 1: Strongly disagree | 2: Disagree | 3: Uncertain | 4: Agree | 5: Strongly agree |
| Comment: | | | | |

| 1. **The space required for the CRP POCT device and operation should be minimal.**   Rationale: As space is at a premium in consultation rooms, the CRP POCT device should be compact. | | | | |
| --- | --- | --- | --- | --- |
| 1: Strongly disagree | 2: Disagree | 3: Uncertain | 4: Agree | 5: Strongly agree |
| Comment: | | | | |

| 1. **Results should be stored on the CRP POCT.**   Rationale: Results should be stored on the CRP POCT device as a backup and for audit purposes. | | | | |
| --- | --- | --- | --- | --- |
| 1: Strongly disagree | 2: Disagree | 3: Uncertain | 4: Agree | 5: Strongly agree |
| Comment: | | | | |

| 1. **CRP POCT results should be automatically transferred from the device to patients’ records.**   Rationale: The CRP POCT results should be transferred from the device to the patient’s medication record to ensure adequate record-keeping. | | | | |
| --- | --- | --- | --- | --- |
| 1: Strongly disagree | 2: Disagree | 3: Uncertain | 4: Agree | 5: Strongly agree |
| Comment: | | | | |

## Section 5: User operation of CRP POCT

| 1. **Minimal staff training for GPs, community pharmacists and advanced nurse practitioners should be required to use the CRP POCT device.**   Rationale: Staff training should be provided to ensure accuracy and familiarity for the use of the device; however, CRP POCT device operation and CRP value interpretation should involve minimal training. | | | | |
| --- | --- | --- | --- | --- |
| 1: Strongly disagree | 2: Disagree | 3: Uncertain | 4: Agree | 5: Strongly agree |
| Comment: | | | | |

| 1. **The time required by staff to run the CRP POCT should be minimal.**   Rationale: Staff time will be required for training, maintenance of equipment, quality control of the device, conducting the CRP POCT and recording of the results. | | | | |
| --- | --- | --- | --- | --- |
| 1: Strongly disagree | 2: Disagree | 3: Uncertain | 4: Agree | 5: Strongly agree |
| Comment: | | | | |

| 1. **Staff operation of the CRP POCT should follow the steps outlined by the manufacturer of the POCT device being used.**   Rationale: Manufacturing instructions potentially differ depending on the individual device being used. | | | | |
| --- | --- | --- | --- | --- |
| 1: Strongly disagree | 2: Disagree | 3: Uncertain | 4: Agree | 5: Strongly agree |
| Comment: | | | | |

| 1. **Maintenance, calibration and quality control will be required for the CRP POCT device as per manufacturer recommendations.**   Rationale: Training should be provided for maintenance of the device (*e.g.,* cleaning, software updates) and quality control procedures. | | | | |
| --- | --- | --- | --- | --- |
| 1: Strongly disagree | 2: Disagree | 3: Uncertain | 4: Agree | 5: Strongly agree |
| Comment: | | | | |

| 1. **Appropriately trained staff in general practice and community pharmacies should be responsible for the quality control of the CRP POCT.**   Rationale: Training should be provided for maintenance of the device (*e.g.,* cleaning, software updates) and quality control procedures. Having other staff members involved in the quality control of the CRP POCT should reduce the workload/pressure on GPs. | | | | |
| --- | --- | --- | --- | --- |
| 1: Strongly disagree | 2: Disagree | 3: Uncertain | 4: Agree | 5: Strongly agree |
| Comment: | | | | |
