# Supplementary material for: Point-of-caRE DiagnostICs for respiraTOry tRact infectionS (PREDICTORS) study: developing guidance for using C-reactive protein point-of-care tests in the management of lower respiratory tract infections in primary care using a Delphi consensus technique
Source: BMJ Open. 2025 May 27;15(5):e101438. doi: 10.1136/bmjopen-2025-101438 (PMC12121597; doi:10.1136/bmjopen-2025-101438)
Supplement: online supplemental file 3 [file bmjopen-15-5-s003.docx]

# Supporting Information File 3: Round 2 Questionnaire

## Criteria fundamentally accepted but wording slightly changed (n = 15)

### Section 1: Using CRP POCT

| **1. A thorough clinical assessment of the patient’s history (e.g., comorbidities, previous admissions), risk profile, and acute clinical situation should precede CRP POCT.**  Rationale: Patients' comorbidities and sources of vulnerability/risk should be considered in conjunction with CRP-based prescribing recommendations before deciding on treatment (1, 2). | | | | |
| --- | --- | --- | --- | --- |
| 1: Strongly disagree | 2: Disagree | 3: Uncertain | 4: Agree | 5: Strongly agree |
| Comment: | | | | |

| **2. Validated, evidence-based clinical decision rules (e.g., STARWAVe and CRB-65) should be incorporated into the clinical assessment of patients suspected of having a LRTI.**  Rationale: Clinical decision rules may provide confirmation of a bacterial LRTI and its severity and helps to identify patients requiring further referral. | | | | |
| --- | --- | --- | --- | --- |
| 1: Strongly disagree | 2: Disagree | 3: Uncertain | 4: Agree | 5: Strongly agree |
| Comment: | | | | |

| **3. CRP POCT should be used for patients suspected of having a LRTI only when the prescriber is uncertain about prescribing antibiotics for LRTIs following their clinical assessment of the patient.**  Rationale: CRP POCT can help reduce inappropriate antibiotic prescribing (1-11). A CRP POCT can give more certainty about the presence/absence of a bacterial LRTI and guide the decision to prescribe/avoid an antibiotic (2). | | | | |
| --- | --- | --- | --- | --- |
| 1: Strongly disagree | 2: Disagree | 3: Uncertain | 4: Agree | 5: Strongly agree |
| Comment: | | | | |

| **4. CRP POCT should aid appropriate prescribing of antibiotics for LRTIs by reducing diagnostic uncertainty as part of the clinical assessment.**  Rationale: CRP is a sensitive and non-specific marker for inflammation which can be used to assess the severity of an inflammation and to predict if an infection can be expected to be self-limiting or severe (2). Uncertainty about the diagnosis of infection can lead to inappropriate antibiotic prescribing (12). CRP POCT helps to assess bacterial LRTI severity and to identify the patients who may benefit from antibiotics (1-4). | | | | |
| --- | --- | --- | --- | --- |
| 1: Strongly disagree | 2: Disagree | 3: Uncertain | 4: Agree | 5: Strongly agree |
| Comment: | | | | |

| **5. Consent, either written or implied, should be obtained from the patient or the patient’s parent or legal guardian in the case of children below the age of 16 for their blood sample to be taken for CRP POCT. Additionally, where appropriate, children should provide their assent.**  Rationale: As with all diagnostic tests, consent will be required for CRP POCT. | | | | |
| --- | --- | --- | --- | --- |
| 1: Strongly disagree | 2: Disagree | 3: Uncertain | 4: Agree | 5: Strongly agree |
| Comment: | | | | |

| **7. CRP POCT should be performed within general practice by general practitioners (GPs), guided by clinical judgement and current best practice guidance.**  Rationale: Patients frequently attend general practice with symptoms of LRTIs; therefore, using a CRP POCT in this setting should help to ensure appropriate prescribing of antibiotics. | | | | |
| --- | --- | --- | --- | --- |
| 1: Strongly disagree | 2: Disagree | 3: Uncertain | 4: Agree | 5: Strongly agree |
| Comment: | | | | |

| **13. CRP POCT should be used in conjunction with a rapid viral test (e.g., SARS-Cov-2/Influenza A&B/RSV Adenovirus antigen combo rapid test) in the presence of cough and fever during an active pandemic or epidemic (such as COVID-19 or Influenza A&B). This is contingent upon the tests demonstrating appropriate sensitivity, specificity, and suitability for the context and population.**  Rationale: In this instance, a rapid viral test could be used to aid interpretation of CRP POCT results (2). For patients that have tested positive for COVID-19, CRP POCT can be useful for disease prognosis (2, 3); in these patients, raised CRP >40 mg/L is indicative of severe infections, indicating the need for close follow-up or hospitalisation (13). | | | | |
| --- | --- | --- | --- | --- |
| 1: Strongly disagree | 2: Disagree | 3: Uncertain | 4: Agree | 5: Strongly agree |
| Comment: | | | | |

### Section 2: The detection of bacterial LRTIs using CRP POCT and the provision of antibiotics

| **17. A CRP value >20 mg/L may indicate the possibility of a bacterial LRTI, though clinical judgement should be used as CRP levels can also be elevated in viral infections (although CRP elevations due to viral infections typically remain below 20 mg/L).**  Rationale: In patients without infection or morbidities that contribute to elevated CRP, serum CRP values are below 5 mg/L (14). Raised serum CRP values often occur in bacterial infections; however, typically only minor elevations are observed in viral infections (5). | | | | |
| --- | --- | --- | --- | --- |
| 1: Strongly disagree | 2: Disagree | 3: Uncertain | 4: Agree | 5: Strongly agree |
| Comment: | | | | |

| **18. A CRP value <20 mg/L may indicate a self-limiting infection (bacterial or viral) for which antibiotics should not be prescribed, though clinical judgement and patient-specific factors should be considered, with clear communication of self-care and re-referral advice to the patient.**  Rationale: Self-limiting infections (viral or bacterial) are those that tend to resolve themselves without further treatment and represent the majority of LRTIs seen in primary care (2). Antibiotics should be withheld when the clinical assessment rules out a severe infection and CRP value is <20 mg/L (1-3, 11). Instead self-care advice can be given and patients advised to re-consult if symptoms worsen or persist (1, 2). | | | | |
| --- | --- | --- | --- | --- |
| 1: Strongly disagree | 2: Disagree | 3: Uncertain | 4: Agree | 5: Strongly agree |
| Comment: | | | | |

| **20. CRP values >100 mg/L in adults indicates the presence of a severe infection for which antibiotics should be prescribed following national/international antibiotic prescribing guidance and hospital referral considered alongside thorough clinical assessment, with attention to the clinical context, severity of illness, and potential non-bacterial causes of elevated CRP levels.**  Rationale: It is strongly recommended to start treatment with antibiotics due to a high risk of a severe bacterial infection at CRP value >100 mg/L (1-3) (pneumonia likely if one of the following are present: new focal chest signs, dyspnoea, tachypnoea, pulse rate >100, fever >4 days (11). Consider hospital referral alongside thorough clinical assessment if CRP value >100 mg/L (2). | | | | |
| --- | --- | --- | --- | --- |
| 1: Strongly disagree | 2: Disagree | 3: Uncertain | 4: Agree | 5: Strongly agree |
| Comment: | | | | |

| **21. CRP values >75 mg/L in children indicates the presence of a severe infection for which antibiotics should be prescribed following national/international antibiotic prescribing guidance and hospital referral considered alongside thorough clinical assessment, with attention to the clinical context, severity of illness, and potential non-bacterial causes of elevated CRP levels.**  Rationale: It is strongly recommended to start treatment with antibiotics due to a high risk of a non-self-limiting infection or refer to hospital (1, 15). Consider urgent hospital referral alongside thorough clinical assessment if CRP value >100 mg/L (1). | | | | |
| --- | --- | --- | --- | --- |
| 1: Strongly disagree | 2: Disagree | 3: Uncertain | 4: Agree | 5: Strongly agree |
| Comment: | | | | |

| **23. For CRP values between 20-100 mg/L in patients with a higher risk of deterioration (i.e., due to existing conditions or severe symptoms), antibiotic prescribing following national/international antibiotic prescribing guidance should be considered, with attention to the clinical context, severity of illness, and potential non-bacterial causes of elevated CRP levels.**  Rationale: For CRP values between 20-100 mg/L, prescribing of antibiotics should be considered in patients with relevant comorbidities, such as chronic obstructive pulmonary disease (exhibiting exacerbation with obvious increased purulence of sputum), diabetes and in vulnerable older patients (2). | | | | |
| --- | --- | --- | --- | --- |
| 1: Strongly disagree | 2: Disagree | 3: Uncertain | 4: Agree | 5: Strongly agree |
| Comment: | | | | |

### Section 4: Features and performance of the CRP POCT device

| **35. Results should be stored on the CRP POCT device or in the patient’s record, with consideration for ease of use and integration into clinical workflow, while ensuring patient privacy and data security.**  Rationale: Results should be stored on the CRP POCT device as a backup and for audit purposes. | | | | |
| --- | --- | --- | --- | --- |
| 1: Strongly disagree | 2: Disagree | 3: Uncertain | 4: Agree | 5: Strongly agree |
| Comment: | | | | |

| **36. CRP POCT results should be automatically transferred from the device to patients’ records where feasible and efficient, leveraging fully integrated electronic healthcare records or easily scannable formats, to streamline documentation and ensure accurate record-keeping.**  Rationale: The CRP POCT results should be transferred from the device to the patient’s medication record to ensure adequate record-keeping. | | | | |
| --- | --- | --- | --- | --- |
| 1: Strongly disagree | 2: Disagree | 3: Uncertain | 4: Agree | 5: Strongly agree |
| Comment: | | | | |

### Section 5: User operation of CRP POCT

| **40. Maintenance, calibration and quality control will be required for the CRP POCT device as per manufacturer recommendations, with support and oversight provided by CRP POCT device supplier (e.g., device manufacturer).**  Rationale: Training should be provided for maintenance of the device (*e.g.,* cleaning, software updates) and quality control procedures. | | | | |
| --- | --- | --- | --- | --- |
| 1: Strongly disagree | 2: Disagree | 3: Uncertain | 4: Agree | 5: Strongly agree |
| Comment: | | | | |

## Criteria that required further consideration (n = 9)

### Section 1: Using CRP POCT

| **8. With appropriate training for the clinical assessment of patients suspected of having a LRTI, healthcare professionals in general practice, other than GPs themselves (i.e., advanced nurse practitioners, general practice pharmacists) should be able to obtain and interpret CRP POCT results with GP oversight.**  Rationale: Having other healthcare professionals involved in diagnosing and managing LRTIs should reduce the workload/pressure on GPs (16). | | | | |
| --- | --- | --- | --- | --- |
| 1: Strongly disagree | 2: Disagree | 3: Uncertain | 4: Agree | 5: Strongly agree |
| Comment: | | | | |

| **9. With appropriate training for treating patients suspected of having a LRTI, healthcare professionals in general practice other than GPs themselves (i.e., advanced nurse practitioners, general practice pharmacists) should be able to act upon CRP POCT results (i.e., prescribe antibiotic therapy when indicated following national/international antibiotic prescribing guidance or provide self-care advice to patients) with GP oversight.**  Rationale: Having other healthcare professionals involved in diagnosing and managing LRTIs should reduce the workload/pressure on GPs (16). | | | | |
| --- | --- | --- | --- | --- |
| 1: Strongly disagree | 2: Disagree | 3: Uncertain | 4: Agree | 5: Strongly agree |
| Comment: | | | | |

| **10. CRP POCT should be performed within community pharmacies by community pharmacists who have received appropriate training, adhere to approved protocols, and liaise with or refer to the patient’s GP when necessary.**  Rationale: Community pharmacists are accessible in a timely manner and regularly provide self-care advice for minor ailments including LRTIs. | | | | |
| --- | --- | --- | --- | --- |
| 1: Strongly disagree | 2: Disagree | 3: Uncertain | 4: Agree | 5: Strongly agree |
| Comment: | | | | |

| **11. With appropriate training for the clinical assessment of patients suspected of having a LRTI, community pharmacists should be able to obtain and interpret CRP POCT results, provided they liaise with or refer to the patient’s GP when necessary.**  Rationale: Community pharmacists are accessible in a timely manner. Enabling them to use CRP POCT in the management of LRTIs should improve patient experience and reduce pressure on general practice . | | | | |
| --- | --- | --- | --- | --- |
| 1: Strongly disagree | 2: Disagree | 3: Uncertain | 4: Agree | 5: Strongly agree |
| Comment: | | | | |

| **12. With appropriate training for treating patients suspected of having a LRTI, community pharmacists should be able to act upon CRP POCT results (i.e., prescribe antibiotic therapy when indicated following national/international antibiotic prescribing guidance or provide self-care advice to patients), provided they liaise with or refer to the patient’s GP when necessary.**  Rationale: Community pharmacists are accessible in a timely manner. Enabling them to use CRP POCT in the management of LRTIs should improve patient experience and reduce pressure on general practice . | | | | |
| --- | --- | --- | --- | --- |
| 1: Strongly disagree | 2: Disagree | 3: Uncertain | 4: Agree | 5: Strongly agree |
| Comment: | | | | |

### Section 2: The detection of bacterial LRTIs using CRP POCT and the provision of antibiotics

| **19. In cases where a child is presented early in the progression of symptoms (i.e., in the first 24 hours), CRP results should be interpreted carefully, with attention to the clinical context and severity of illness. In this instance, a CRP value <5 mg/L may indicate a self-limiting infection (bacterial or viral) for which antibiotics should not be prescribed.**  Rationale: Parents of unwell children typically present early with their child in primary care. In these cases, CRP POCT results should be interpreted carefully (1). Various CRP thresholds for withholding antibiotics in children have been reported (<20 mg/L (1, 17), <10 mg/L (17, 18), or <5 mg/L (15)). | | | | |
| --- | --- | --- | --- | --- |
| 1: Strongly disagree | 2: Disagree | 3: Uncertain | 4: Agree | 5: Strongly agree |
| Comment: | | | | |

| **22. For CRP values between 20-100 mg/L in low-risk patients (i.e., not at a higher risk of deterioration due to existing conditions or severe symptoms), prescribing of antibiotics should be avoided or delayed following national/international antibiotic prescribing guidance, though clinical judgement and patient-specific factors should be considered, with clear communication of self-care and re-referral advice to the patient.**  Rationale: For CRP values between 20-100 mg/L, the clinical assessment of patients presenting with a LRTI remains the primary decision-driver in whether to prescribe an antibiotic (1, 2). In most cases, antibiotics are not needed. Delayed prescribing can be considered if illness severity did not require immediate antibiotics and CRP value is between 20-100 mg/L (1-5). | | | | |
| --- | --- | --- | --- | --- |
| 1: Strongly disagree | 2: Disagree | 3: Uncertain | 4: Agree | 5: Strongly agree |
| Comment: | | | | |

### Section 3: Communication strategies to increase antibiotic stewardship

| **26. For CRP POCT conducted in community pharmacies, patients should be provided with a detailed description of the assessment undertaken and the results obtained so they can provide these to their GP if necessary.**  Rationale: Depending on clinical assessment and CRP POCT results, patients should either be provided advice on self-care in community pharmacies or referred to general practice for antibiotic prescriptions. CRP POCT results should be sent to the GP for transparency. | | | | |
| --- | --- | --- | --- | --- |
| 1: Strongly disagree | 2: Disagree | 3: Uncertain | 4: Agree | 5: Strongly agree |
| Comment: | | | | |

### Section 5: User operation of CRP POCT

| **37. CRP POCT providers (e.g., GPs, community pharmacists and advanced nurse practitioners) should undergo training to use the POCT device and to interpret CRP results, combined with enhanced communication skills training to effectively convey findings and implications to patients.**  Rationale: Staff training should be provided to ensure accuracy and familiarity for the use of the device; however, CRP POCT device operation and CRP value interpretation should involve minimal training. | | | | |
| --- | --- | --- | --- | --- |
| 1: Strongly disagree | 2: Disagree | 3: Uncertain | 4: Agree | 5: Strongly agree |
| Comment: | | | | |
