# Supplementary material for: Point-of-caRE DiagnostICs for respiraTOry tRact infectionS (PREDICTORS) study: developing guidance for using C-reactive protein point-of-care tests in the management of lower respiratory tract infections in primary care using a Delphi consensus technique
Source: BMJ Open. 2025 May 27;15(5):e101438. doi: 10.1136/bmjopen-2025-101438 (PMC12121597; doi:10.1136/bmjopen-2025-101438)
Supplement: online supplemental file 4 [file bmjopen-15-5-s004.docx]

# Supporting Information File 4: Round 3 Questionnaire

## Criteria requiring further consideration (n = 7)

### Section 1: Using CRP POCT

| **3. CRP POCT should be used for patients suspected of having a LRTI only when the prescriber is uncertain about prescribing antibiotics for LRTIs following their clinical assessment of the patient.** | | | | |
| --- | --- | --- | --- | --- |
| 1: Strongly disagree | 2: Disagree | 3: Uncertain | 4: Agree | 5: Strongly agree |

| **8. With appropriate training for the clinical assessment of patients suspected of having a LRTI, healthcare professionals in general practice, other than GPs themselves (i.e., advanced nurse practitioners, general practice pharmacists) should be able to obtain and interpret CRP POCT results with GP oversight.** | | | | |
| --- | --- | --- | --- | --- |
| 1: Strongly disagree | 2: Disagree | 3: Uncertain | 4: Agree | 5: Strongly agree |

| **9. With appropriate training for treating patients suspected of having a LRTI, healthcare professionals in general practice other than GPs themselves (i.e., advanced nurse practitioners, general practice pharmacists) should be able to act upon CRP POCT results (i.e., prescribe antibiotic therapy when indicated following national/international antibiotic prescribing guidance or provide self-care advice to patients) with GP oversight.** | | | | |
| --- | --- | --- | --- | --- |
| 1: Strongly disagree | 2: Disagree | 3: Uncertain | 4: Agree | 5: Strongly agree |

| **10. CRP POCT should be performed within community pharmacies by community pharmacists who have received appropriate training, adhere to approved protocols, and liaise with or refer to the patient’s GP when necessary.** | | | | |
| --- | --- | --- | --- | --- |
| 1: Strongly disagree | 2: Disagree | 3: Uncertain | 4: Agree | 5: Strongly agree |

| **11. With appropriate training for the clinical assessment of patients suspected of having a LRTI, community pharmacists should be able to obtain and interpret CRP POCT results, provided they liaise with or refer to the patient’s GP when necessary.** | | | | |
| --- | --- | --- | --- | --- |
| 1: Strongly disagree | 2: Disagree | 3: Uncertain | 4: Agree | 5: Strongly agree |

| **12. With appropriate training for treating patients suspected of having a LRTI, community pharmacists should be able to act upon CRP POCT results (i.e., prescribe antibiotic therapy when indicated following national/international antibiotic prescribing guidance or provide self-care advice to patients), provided they liaise with or refer to the patient’s GP when necessary.** | | | | |
| --- | --- | --- | --- | --- |
| 1: Strongly disagree | 2: Disagree | 3: Uncertain | 4: Agree | 5: Strongly agree |

### Section 2: The detection of bacterial LRTIs using CRP POCT and the provision of antibiotics

| **19. In cases where a child is presented early in the progression of symptoms (i.e., in the first 24 hours), CRP results should be interpreted carefully, with attention to the clinical context and severity of illness. In this instance, a CRP value <5 mg/L may indicate a self-limiting infection (bacterial or viral) for which antibiotics should not be prescribed.**  Rationale: Parents of unwell children typically present early with their child in primary care. In these cases, CRP POCT results should be interpreted carefully (1). Various CRP thresholds for withholding antibiotics in children have been reported (<20 mg/L (1, 17), <10 mg/L (17, 18), or <5 mg/L (15)). | | | | |
| --- | --- | --- | --- | --- |
| 1: Strongly disagree | 2: Disagree | 3: Uncertain | 4: Agree | 5: Strongly agree |
