# Supplementary material for: Point-of-caRE DiagnostICs for respiraTOry tRact infectionS (PREDICTORS) study: developing guidance for using C-reactive protein point-of-care tests in the management of lower respiratory tract infections in primary care using a Delphi consensus technique
Source: BMJ Open. 2025 May 27;15(5):e101438. doi: 10.1136/bmjopen-2025-101438 (PMC12121597; doi:10.1136/bmjopen-2025-101438)
Supplement: online supplemental file 5 [file bmjopen-15-5-s005.docx]

# Supporting Information File 5: Round 1 Comments

Please note all comments are verbatim.

## Section 1: Using CRP POCT

1. CRP POCT should be used for all patients suspected of having a lower respiratory tract infection (LRTI) regardless of age or demographics.

| It is possible to withhold antibiotics on clinical grounds in a significant proportion.  May not be appropriate in children, some elderly patients don't mount a CRP response or it is too early in their clinical course to mount one or it may not influence your decision to prescribe which may be dominated by other things e.g. if the patient is immunocompromised.  Should be dependent on patients medical history.  Could help persuade patients too.  I would always consider the clinical context in the first instance. If the test is unlikely to change my management plan, I would not use it.  There can be conflicting evidence on the sensitivity/specificity of some available products. Such testing should have demonstrated benefit in The specific context and patient population to which it is being applied. I would apply this comment to all below questions.  Use should be more targeted.  Use of testing should be based first on assessment and symptoms that might indicate LRTI and not just to all patients presenting.  It will not change mgt in very well patients with LRTI (i.e. mild cough) and those who are systemically unwell i.e. CRB 1 or more.  Agree that in some cases it would be useful, but in mild/self-limiting cases where this is clear from the outset I don't believe all patients should have CRP-POCT. |
| --- |

1. CRP POCT should be used for patients suspected of having a LRTI only when the prescriber is uncertain about prescribing antibiotics for RTIs.

| This seems more reasonable rather than a blanket approach.  But with caveat mentioned.  Can be difficult clinically.  I would always consider the clinical context in the first instance. If the test is unlikely to change my management plan, I would not use it.  Using CRP POCT would provide greater certainty, irrespective of prescriber’s level of certainty.  Perhaps it should be carried out on anyone with a LRTI.  Difficult to be sure on diagnosis of bacterial infection, this will provide additive information to make further decision on.  Yes to support the clinical decision making, as an additional piece of information to consider in the context of the full picture. |
| --- |

1. CRP POCT should be used for patients suspected of having a LRTI when the prescriber is thinking about prescribing antibiotics for LRTIs.

| You may decide to give a backup prescription that will not be influenced by the CRP results.  Following a standardised and validated review.  May stop some unnecessary prescriptions.  I would always consider the clinical context in the first instance. If the test is unlikely to change my management plan, I would not use it. Sometimes I would make the decision and the CRP is unlikely to change it. GPs rely heavily on clinical skills when making decisions and tests are not always needed to aid the decision. Certainly tests can be helpful. Some of the decision making around this depends on detail. For example, if the test takes a long time to process (say any longer than two minutes), then it is less likely to be used. 2 minutes is a significant proportion of the total duration of a consultation.  Much more data is available for a POCT.  'Thinking about' and 'uncertainty' are pretty similar.  If based on assessment and discussion with patient considering antibiotics, this should be provided to further confirm the need as often antibiotics are prescribed unnecessarily based on a short consultation with a GP.  If patient v unwell this test will not change mgt.  Would be useful to back up the prescribing decision. In the event that the CRP is then low (indicating viral infection) it may encourage a prescriber to reconsider and not prescribe antibiotics. |
| --- |

1. CRP POCT results should be interpreted with caution in patients with existing conditions that can elevate CRP values (e.g., arthritis, gout, inflammatory bowel disease) and in those receiving immunotherapy.

| Chronic elevation can cause issues.  Could be raised due to underlying condition.  There are causes of elevated CRP other than bacterial infection. This comment is self-evident to my mind.  I would check guidance before use.  Baseline CRP (from non-infection time period) would be helpful here, but as this is not likely to be available, CRP results should be interpreted cautiously. |
| --- |

1. CRP POCT should reduce diagnostic uncertainty as part of the assessment for LRTIs and aid prescribing decisions.

| Can be elevated in viral infections as well but not as high as bacterial.  Where CRP low.  This is the precise situation where I see CRP POCT having a role.  Probably but not alone without a lot of other data. |
| --- |

1. CRP POCT results should be interpreted in conjunction with a thorough assessment of the patient’s history, risk profile, and acute clinical situation.

| Even if bacterial infection is not present a person can be seriously ill due to viral illness and require supportive therapy. There are two questions in the assessment 1. How ill is someone 2. Do they need antibiotics.  As above - lots of factors go into a decision re: prescribing e.g. co morbidities / previous admissions etc.  Needs clinical correlation.  As before, the CRP test is part of the puzzle. Clinical factors are the main part of the puzzle. There are some instances where the clinical picture give the full picture. There are others where the additional CRP piece of the jigsaw completes the picture.  Testing of all patients presenting with CRP POCT testing without first looking at this could lead to false positives. |
| --- |

1. Clinical decision rules that are proven for use in your practice should be incorporated into the clinical assessment of patients suspected of having a LRTI.

| Patients sometimes have trouble trusting these tools.  FeverPain is used for diagnosis of tonsilitis, not LRTI? 4 that it could be used with CRB65?  FeverPAIN and similar scores (Centor; CRB65) are very helpful in making clinical decisions in my view.  I would be very cautious in using clinical decision rules (e.g. such as FeverPAIN), some of these tools need validation and further research to support the conclusion that they confirm a bacterial LRTI. I agree with the question, but not necessarily the rationale provided with this question which states 'confirmation of a bacterial LRTI'. Incorporating them into the process of clinical assessment would be helpful, with the caveats provided of where these stand in terms of the evidence base and certainty of interpretation. |
| --- |

1. CRP POCT should be performed within general practice.

| Yes we see lots of people with LRTIs.  As long as result available very quickly.  It depends on the evidence and the feasibility. I do not know enough about this to be sure.  Too many other variables to be considered, if extended to include BNP eosinophils, would be of value.  However set up and ongoing cost along with time required for testing may be significant limitations on the practicalities of it being used in a busy GP practice.  GP is an ideal setting to perform POCT once GPs are trained. Having additional information at the point of assessment and prescribing provides further information to support the decision making process. More diagnostics are needed in primary care to help rationalise antibiotic prescribing. |
| --- |

1. Healthcare professionals in general practice, other than general practitioners (GPs) themselves (i.e., advanced nurse practitioners, general practice pharmacists) should be able to obtain and interpret CRP POCT results.

| In use in the UK as such in GP practices.  Need to be cognisant that they must also assess severity of illness as well as whether bacterial or not. Must also be able to identify that this may not be a LRTI e.g. heart failure or pulmonary embolus.  With training.  I would worry about overuse of CRP if it is deployed widely. I see this in my clinical practice. PSAs are done by other staff that I definitely would not have done, but I am left picking up the pieces. Maybe as evidence accrues more widespread use would be appropriate. For the moment I am strongly of the view that it should be confined to use by the individual who ultimately has clinical responsibility.  Other healthcare practitioners mentioned would still be under the governance of the GP. I am not convinced this will reduce workload of GPs as we will still have to supervise others which can be more difficult than if do assessment ourselves. I have not seen levels of experience or training by other healthcare professionals specifically in this domain yet to feel confident that this is appropriate as things stand.  Same as above, it is not a question of who but what.  With training and education, if evidence based - as for GPs.  Comorbidities may complicate the diagnosis (e.g. other inflammatory conditions) and this information may not be available to others HCPs. Additionally, other duties and current workload may impact on the potential for other HCPs to diagnose.  Use of other HCP in other clinical settings could be of benefit to patients and also reduce workload on GPs by delegating tasks to other HCP that they might also be able to deliver.  With appropriate training and processes, any HCP in GP can perform and interpret POCT. However, the decision to prescribe an antibiotic needs be the responsibility of a HCP who is conducting the clinical assessment, prescribing etc. At present this is largely the GP in the Irish context. Moving this into the domain of other HCPs (e.g. nurses, pharmacists) would reduce GP workload, but this should not be a primary driver here, AMS (stewardship) needs to be the primary focus at all times. If other HCPS are involved, there needs to be consideration given to AMS. |
| --- |

1. Healthcare professionals in general practice, other than GPs themselves (i.e., advanced nurse practitioners, general practice pharmacists) should be able to act upon CRP POCT results (i.e., prescribe antibiotic therapy when indicated following national/international antibiotic prescribing guidance or provide self-care advice to patients).

| See caution in question 15 also.  Depending on training.  Would need clear protocols on this and are they trained to listen to chest?  ABX prescribing decisions depend on a full clinical assessment, not just a CRP result. I would worry that liberalising antibiotic prescribing will make our antimicrobial resistance problem worse.  As above answer - not with the current lack of specific training in this area but may be possible in future. However, GPs workload won't reduce if they have to supervise this along with their regular duties which continue to expand and expand in modern healthcare.  If the necessary education on how to complete other diagnostic tests are implemented.  Need to see evidence on that.  Comorbidities may complicate the diagnosis (e.g. other inflammatory conditions) and this information may not be available to others HCPs. Additionally, other duties and current workload may impact on the potential for other HCPs to act upon/ recommend treatments.  To ensure it continues to reduce the workload and to add value to the patient the HCP providing the service should be able to act on the result.  As per Q 15 comment. With equal emphasis given to self-care advice as well as an accepted management strategy for infections that do not require an antibiotic. |
| --- |

1. The decision whether to perform CRP POCT and whether to prescribe antibiotics should remain at the discretion of the GP.

| Sensitivity of CRP is not sufficient to exclude need for antibiotics on its own.  If there is a clear guideline / protocol then I think others could do it e.g. ANP.  As we learn more it perhaps could be liberalised, but for the moment I think it should be restricted to the practitioner with ultimate clinical responsibility.  I think other the interpretation of diagnostic tests and gatekeeping of antimicrobials should be devolved to other clinicians. This is particularly important in the Irish context where GPs frequently report overburden, burnout and where it may be difficult for patients to get GP appointments.  Clinical judgement is always part of an assessment and should not be disregarded ‘treat the patient not the test’.  Until I see evidence supporting alternative approach.  Mandatory practice would be best as it would be implemented consistently and better diagnoses would result.  There should still be some autonomy in the decision to prescribe by the GP, as each patient is different. There could be clear guidelines however which should be in the majority of cases followed, similarly to other clinical conditions but where also prescribing outside this is possible with good clinical reasoning.  As previous comments, the AMS agenda and the reduce GP workload agenda may come head to head here. The main driver to appropriate antibiotic use should be AMS - at present in Ireland I do not believe we have another HCP workforce available in general practice who is skilled in clinical assessment and has prescribing authority. Enhanced training on RTI assessment, patient review in conjunction with their history and co-morbidities, would be needed before nurses/pharmacists potentially move into this space. I am thinking of the co-morbid older adult, with significant history, presenting with RTI/pneumonia/IECOPD, where would the responsibility of care rest when it comes to assessment and prescribing? |
| --- |

1. CRP POCT should be performed within community pharmacies.

| Would depend on if the pharmacist could also prescribe antibiotics or not prescribing antibiotics for LRTIs may not be the best one to start with.  CRP shouldn’t be used alone; a full consultation should be needed. Until pharmacists are paid for such a service I don’t believe it should be offered.  Would require further training on diagnosis of conditions other than LRTI.  What would happen if the result was elevated? Would this generate a GP apt?  An education piece needs to be done around the selection, use and validation of medical devices.  As we learn more it perhaps could be liberalised, but for the moment I think it should be restricted to the practitioner with ultimate clinical responsibility.  I think this would be best placed for pharmacists within GP practices or for pharmacists within specific clinics nested within community pharmacies. For a dispensary-based pharmacist with a busy workload, this may be challenging.  Needs evidence base.  It would be another useful point of testing but would need to be in conjunction with GP due to current practices.  With the available consultation room space, clinical expertise and resources available in the community pharmacy, there should be no issue with providing CRP POCT in the pharmacy by trained HCP. To ensure though the value of the service the pharmacist should be able to interpret and act on the result.  Community pharmacists can support POCT, and can be trained to roll out POCT as a clinical service. As per previous comments, clarity of where the responsibility would lie in terms of clinical assessment (including physical assessment) is important to consider as part of this. |
| --- |

1. Appropriately trained community pharmacists should be able to obtain and interpret CRP POCT results.

| Until pharmacists can prescribe on foot of these results I don’t know how much value it will add.  I don’t believe it is wise to widen antibiotic prescribing. This has not gone well in some southern European countries. Ireland has high DDD ABX prescribing compared to the rest of Europe. Also: clinical examination of the chest is not a part of pharmacists’ training.  Reducing GP workload depends on the sensitivity and specificity of the test. Appropriate training is not the same as clinical experience.  Yes, once fully trained. Comorbidities remains a potential complicating factor that needs consideration.  Pharmacists have good experience of the use of POCT and following best practice guidelines, CPD requirements and training would ensure that pharmacists keep up to date with any service provided by them in their pharmacy. By ensuring the pharmacist can follow through on the results will ensure that the patient has a better experience in the health system, a faster response time without waiting for a GP consultation and can be provided evidence based information on what action to take. This should reduce the number of patients unnecessarily visiting the GP practice. |
| --- |

1. Appropriately trained community pharmacists should be able to act upon CRP POCT results (i.e., prescribe antibiotic therapy when indicated following national/international antibiotic prescribing guidance or provide self-care advice to patients).

| If pharmacists are paid for a full respiratory consult, according to appropriate training, guidelines and prescribing rights I think this could be beneficial but safety netting for GP/ hospital referral would need to be clear.  GP would still need to do clinical review/assessment of chest if prescribing antibiotics.  Certified training should be in place.  I don’t believe it is wise to widen antibiotic prescribing. This has not gone well in some southern European countries. Ireland has high DDD ABX prescribing compared to the rest of Europe. The CRP test is one aspect to the clinical assessment. Examining lungs is outside of the scope of pharmacy practice. We need to reduce our antibiotic prescribing.  As above, comorbidities need to be considered.  Pharmacists make clinical decisions in their practice all the time, with the expanding role of the pharmacist, prescribing pharmacists should become a part of community pharmacy practice. Being able to act on the results of POCT, ensures patients who need antibiotics access these in a timely manner and without the additional need of another visit to another HCP which could take days to arrange and delay the patients treatment.  As per previous comment. Where CRP POCT could be very useful in pharmacy is to support a viral infection/self-management pathway of care. However, if the CRP level is high, then clear guidance on what steps to take next are needed e.g. communication with GP, GP referral, consideration of patient recent infection/antibiotic history, comorbidities. |
| --- |

1. Group A strep testing for patients presenting with a sore throat is more suitable for use within community pharmacies than CRP POCT for LRTIs.

| This is more straightforward condition as unlikely to be other illnesses. Pneumonia is frequently misdiagnosed.  Could reduce presentation to GPs for viral infections.  A clinical assessment is more important that GAS testing. Detecting GAS does not mean an antibiotic is needed. It means an antibiotic MIGHT be needed. Most sore throats (GAS or viral) resolve without ABX.  Needs evidence – could also have different effect – overuse in earlier presentations.  Both could potentially be used to equal effect. It would require legislative changes and guideline implementation to be workable and to protect pharmacists.  Both provide an opportunity in community pharmacy to provide evidence based guidance to patients about their treatment and to reduce unnecessary antibiotic use. Both throat swaps and blood sampling require clinical skills in a consultation room and screening of appropriate patients (not testing all patients presenting at the pharmacy).  There is potentially more suitability for Group A strep testing, as this would be for a minor illness. Evidence has shown that this system can be rolled out in pharmacy and promote appropriate antibiotic use. |
| --- |

1. The decision whether to perform CRP POCT should remain at the discretion of the community pharmacist.

| I don’t believe pharmacists should prescribe antibiotics without a POCT indication.  However may be pressurised by commercial interests.  I would worry about overuse of CRP if it were deployed widely. I see this in my clinical practice. PSAs are done by other staff that I definitely would not have done, but I am left picking up the pieces. Maybe as evidence accrues more widespread use would be appropriate. For the moment I am strongly of the view that it should be confined to use by the individual who ultimately has clinical responsibility.  I think that this places the pharmacist at risk.  If implemented it should be implemented fully across all practice settings (i.e. gp and pharmacy settings). It should be patient-focused rather that HCP-focussed.  Guidelines should be in place to determine which patients should receive CRP POCT and this should be interpreted by the pharmacist, not all patients who request CRP POCT should be provided with it as this could lead to inaccurate results.  In some cases, e.g. if a patient requested it, or a GP/OOH/nursing home requested it, would the pharmacist run the test? |
| --- |

1. Following clinical assessment of patients presenting with symptoms of a LRTI, GPs should refer patients to community pharmacy for CRP POCT with a prescription on-hold for antibiotics.

| I would not be confident with doing this , the prescribe who ever that is should have access to CRP to help them with their prescribing decision at the point of prescribing, I would not be happy with this as either a health professional or a patient.  I feel a consultation should be started and finished by one Healthcare Professional- who is ultimately responsible for the patient in this scenario?  Interesting concept and could work!  Sounds like a novel idea – hadn’t thought of this.  GP will most likely want to make clinical decision with CRP POCT and if pharmacist does this on GP behalf, there may be patient pressures to dispense?  If pharmacists would want this?  Fragmentation of care is a terrible idea. It introduces clinical risk and communication problems.  I’m not sure this is the best use of time for the clinicians involved, or the patient.  I would not engage with CRP POCT if not available to do within the consultation – in real life this model will only increase workload – back and forth emails/phone calls to pharmacist /borderline results etc.  Why can’t GP just do the POCT – or a practice-based pharmacist?  Both professions should be involved to share the workload. If pharmacists are taking on such workload then remuneration and guideline generation need implementation.  This could be one route into testing and evidence based decision making but for a patient and to obtain a timely response may not be the best.  This is a difficult scenario, if a prescription is already issued, even without the CRP level, what real impact would a low CRP level have if a patient already has the prescription. Ideally, the CRP level should be available first and considered before considering a prescription, delayed or otherwise. |
| --- |

1. Consent will be provided by the patient or the patient’s parent or legal guardian in the case of children below the age of 16 for their blood sample to be taken for CRP POCT.

| Yes essential otherwise it is assault.  In addition to parental consent, the child would need to assent if I am to do the test. I certainly would not want to have a parent restrain a child whilst I take the test.  Wow lots of burden.  But not necessarily written consent – it is implied. |
| --- |

1. CRP POCT should be used in conjunction with a rapid viral test (e.g., SARS-Cov-2/Influenza A&B/RSV Adenovirus antigen combo rapid test) in the presence of cough and fever during an active pandemic or epidemic (such as COVID-19 or Influenza A&B).

| With a caveat that a secondary bacterial infection may present post viral infection which may necessitate antibiotic treatment.  However limited panel. RSV major issue in elderly also. Starts to become more complicated!  Can get bacterial co infection.  It depends on the performance of the Ag test and the speed it takes to develop it. I would also have some concerns about exposing practice staff to increased risk of viral illness transmission. This situation really depends on the detail.  Yes, again similar to my comment in the first question, there should be demonstrated sensitivity and specificity of the test and suitability for the context and population to which the test(s) is applied.  Needs evidence but seems reasonable but may not be cost effective. |
| --- |

1. The cost of the CRP POCT device should be reimbursed by Governments.

| Improved antibiotic prescribing will save the government in the long run.  Might not be feasible for practices to buy themselves.  Would pilot privately, room for abuse.  It depends on the cost-effectiveness/HTA, but assuming it is cost effective, the HSE should support the testing.  Should be reimbursed as a public health priority.  If working as part of a National Health Strategy, the funding should be present to ensure patients can all access this treatment for decision making and not just those who can afford to pay for it. |
| --- |

1. The cost of the CRP POCT consumables should be reimbursed by Governments.

| It depends on the cost-effectiveness/HTA, but assuming it is cost effective, the HSE should support the testing.  Should be reimbursed as a public health priority. |
| --- |

1. CRP POCT providers (i.e., GPs and/or community pharmacists) should be reimbursed by Governments.

| It depends on the cost-effectiveness/HTA, but assuming it is cost effective, the HSE should support the testing.  This requires extra work and if it reduces hospital referrals or use of antibiotics will need resourcing to make it viable.  Extra test/ extra time/ extra training.  Would take time to perform.  If it is quick and easy to do such as blood glucose testing then I would not expect reimbursement for my time but would expect it to pay for the device and consumables.  Benefit is to the health system and for population health.  Providing POCT requires time, space, it also takes away from other workloads and HCP must be reimbursed for this to ensure that a high standard is provided and where needed additional HCP or support staff can be hired. |
| --- |

## Section 2: The detection of bacterial LRTIs using CRP POCT and the provision of antibiotics

1. CRP POCT results can inform the need for antibiotic therapy in LRTIs.

| Need to take the whole picture in consideration.  A complete assessment is required rather than looking at the CRP result in isolation.  Full clinical assessment remains important particularly with comorbid inflammatory conditions. |
| --- |

1. A CRP value >20 mg/L indicates the possibility of a bacterial LRTI.

| CRP can be high with viral infections.  I am not familiar with cut offs that define or rule out the possibility of a bacterial infection.  With the note that borderline or mildly elevated values may be more difficult to interpret. Need to consider if patient is taking immunosuppressive therapy which may impact CRP, cancer diagnoses etc. |
| --- |

1. A CRP value <20 mg/L indicates a self-limiting infection (bacterial or viral) for which antibiotics should not be prescribed.

| In adults.  However CRP not static , can increase rapidly.  Assuming the premise is correct, ABX should be avoided in self-limiting illness be it bacterial or viral.  I would have a little concern about using absolute cutoff values. Self-care and re-referral advice should be made clear to the patient, as well as the uncertainties around the result.  Unless other inflammatory condition/ immunosuppressed etc. Has to be interpreted in context of full assessment. |
| --- |

1. CRP POCT should have a lower threshold for withholding antibiotics in children (<5 mg/L or <10 mg/L) than adults (<20 mg/L).

| I'm not too familiar with CRP thresholds in children. Ideally no blood test should be done in young children.  I am not familiar with cut offs that define or rule out the possibility of a bacterial infection in children.  I would like to look at the full rigour and quality of the reporting evidence before making a decision.  The CRP test may not be acceptable to some children. |
| --- |

1. CRP values >100 mg/L in adults indicates the presence of a severe infection for which antibiotics should be prescribed following national/international antibiotic prescribing guidance and hospital referral considered alongside thorough clinical assessment.

| Can happen for things other than bacterial infection.  Again, the clinical context is important here too, but of course referral is more likely to be needed in these circumstances. In addition, account would need to be taken of the ceiling of care. |
| --- |

1. CRP values >75 mg/L in children indicates the presence of a severe infection for which antibiotics should be prescribed following national/international antibiotic prescribing guidance and hospital referral considered alongside thorough clinical assessment.

| Again, the clinical context is important here. Severe illness needs referral to hospital, of course. |
| --- |

1. For CRP values between 20-100 mg/L in low-risk patients (i.e., not at a higher risk of deterioration due to existing conditions or severe symptoms), prescribing of antibiotics should be avoided or delayed following national/international antibiotic prescribing guidance.

| Need for underlying factors to be considered.  Low risk patients every effort should be made to avoid prescribing antibiotics. I am not aware of specific cut offs regarding the use of CRP.  I would query if patients would actually delay antibiotic prescribing. If they have been told the CRP is 44 for example, they may look this up online and then decide they need the prescription. |
| --- |

1. For CRP values between 20-100 mg/L in patients with a higher risk of deterioration (i.e., due to existing conditions or severe symptoms), antibiotic prescribing following national/international antibiotic prescribing guidance should be considered.

| With any infection antibiotics should be considered. In a small proportion of such cases, where they are likely to be of benefit, they should be prescribed.  A point in the process to consider the individual patient history, risk factors etc. is key and vital to this. The values and an algorithm is helpful, but other interventions and supports such as good communication, discussion of self-management, safety-netting, potential for molecular diagnostics, need to also be considered. |
| --- |

## Section 3: Communication strategies to increase antibiotic stewardship

1. Patients should be informed about antibiotic resistance, the role (or lack thereof) of antibiotics in treating LRTIs, and antibiotic stewardship.

| Time limits in a consultation may prevent this - an information leaflet to give to the patient may help.  Very important for the environment/resistance/often not what patients want to hear.  I think a strong, multimedia education campaign is absolutely necessary.  This needs to be balanced to ensure that those who require and start antibiotics complete the full course and are not at risk of mixed-messaging which could result in taking partial courses of treatment.  I believe patients should always be told of the risks of resistance, not just the short-time ADRs e.g. GI symptoms. This messaging needs to happen every time an infection or antibiotic is being considered. |
| --- |

1. CRP POCT results can be used to support patient-healthcare professional communication, especially when explaining whether antibiotics are required for a LRTI.

| Depends on their positive predictive value.  Yes, a helpful tool which helps to shine objectivity on decisions and thereby help patient acceptance where antibiotics are not prescribed.  I agree, this will largely need a lot of time on the part of the HCP, trust by the patient in the HCP, and other supports to provide the patient such as information leaflets etc. may be useful. A culture shift in thinking in the population is needed to move to a place where self-management first is more acceptable rather than immediately requesting an antibiotic. |
| --- |

1. For CRP POCT conducted in general practice, outcomes of the test should be provided to community pharmacists.

| How would this communication take place?  Fragmentation of care is risky. Providing the CRP to the pharmacist spreads care around and increases clinical risk.  This is not practical in reality.  Why is this needed - GP can provide this advice if making the assessment?  Yes, and it also helps to reduce antibiotic seeking behaviour as ‘loans’ / ‘emergency supplies’ from community pharmacy where no prescription is present.  This would require a very clear line of communication, it sounds good and worthwhile for pharmacists to know this, but I struggle to see how this would be implemented. If the GP has conducted the test, then they should also provide self-care advice. If the management plan is involving an initial GP consultation followed by pharmacist consultation/review, it is then requiring input from two HCPs, more time and resourcing? I am not convinced on the specifics of how this would work I will be honest. |
| --- |

1. For CRP POCT conducted in community pharmacies, outcomes of the test should be provided to GPs.

| Results should only be sent if patient needs to attend GP and if so should be given to patient to bring to GP. Transmitting results without clinical context and without patient being able to access GP is medicolegally dangerous.  This would inevitably generate more work for GPs - to review and interpret results. There would need to be an agreement beforehand/dedicated time.  A CRP without a clinical assessment is not very helpful to the GP. It might be.  We are swamped with paperwork - this would not be helpful and creates work.  For patient record.  I agree that in all cases the CRP level should be communicated with the GP who has responsibility for the patient, there could be an automatic method for this but also as per Q39 the implementation of this is important. I believe this is important as if the patient deteriorates/does not improve, the GP knowledge of a CRP test is important. |
| --- |

1. CRP POCT should be used together with enhanced communication skills training.

| Clinicians sometimes prescribe antibiotics when they know they are unlikely to help. This is a reflection of various things, but one item that can assist is high quality communication skills, such as the CHESTSSS consultation model.  Most definitely.  The language around resistance, appropriate antibiotic use for patients is very important. There is a lack of widespread understanding of the risks of inappropriate antibiotic use. |
| --- |

## Section 4: Features and performance of the CRP POCT device

1. CRP POCT results should have a high sensitivity.

| Moderate sensitivity.  In an ideal world, it ‘should’. |
| --- |

1. CRP POCT results should have a high specificity.

| In an ideal world, it ‘should’, but there are causes of an elevated CRP beyond bacterial infection. |
| --- |

1. CRP POCT results should have a high positive predictive value.

| In an ideal world, it ‘should’, but there are causes of an elevated CRP beyond bacterial infection. |
| --- |

1. CRP POCT results should have a high negative predictive value.

| In an ideal world, it ‘should’.  The clinical context is key also possibly old records showing previous CRP results in people with inflammatory conditions. |
| --- |

1. Detection of a possible bacterial LRTI directly from a patient’s blood sample using CRP POCT should be completed in a one-step process.

| Ease of use and a rapid result are crucial for use in general practice.  If possible.  Seems more efficient, depends more on how long it takes to get the result.  Reduces human error. |
| --- |

1. CRP POCT should require a small sample volume of blood.

| Yes, in an ideal world.  Spot blood sample if possible. |
| --- |

1. The space required for the CRP POCT device and operation should be minimal.

| Yes, preferably. |
| --- |

1. Results should be stored on the CRP POCT.

| It depends on the detail. If the implication of this is that I would have to input the name and DOB of every patient on whom I undertake a CRP test, this would add to time and will reduce the utility of the device.  Or in patient record.  Cloud storage much safer and reliable for various reasons.  Also in patient's PMR.  Not as the only source of record-keeping, appropriate patient review forms/records are also important. |
| --- |

1. CRP POCT results should be automatically transferred from the device to patients’ records.

| Anything that saves time is to be welcomed.  Assuming a fully integrated electronic healthcare record is available.  Not crucial if complex between two systems or time consuming. GP will write in record anyway.  Or easily scanned in.  Yes, helps to avoid oversight and error. |
| --- |

## Section 5: User operation of CRP POCT

1. Minimal staff training for GPs, community pharmacists and advanced nurse practitioners should be required to use the CRP POCT device.

| It should require what is necessary even if a little involved.  Training on use and interpretation of results can be completed in online or distance training. Governance structures in HC practices will take responsibility for standard of POCT in the practice.  'Minimal staff training' suggests very little, I believe this should be combined with communication training and what this means for patients, interpretation, self-management when appropriate etc. |
| --- |

1. The time required by staff to run the CRP POCT should be minimal.

| Less than a minute would be preferable.  Yes, this is desirable as workloads are increasing across all HCP settings.  The test should provide a result quickly, but staff need to factor in time to discuss this carefully with the patient. |
| --- |

1. Staff operation of the CRP POCT should follow the steps outlined by the manufacturer of the POCT device being used.

| N/A |
| --- |

1. Maintenance, calibration and quality control will be required for the CRP POCT device as per manufacturer recommendations.

| Maintenance by the manufacturer is always more expert, professional and reliable. Expertise is important here and manufacturers would be best placed. |
| --- |

1. Appropriately trained staff in general practice and community pharmacies should be responsible for the quality control of the CRP POCT.

| HSE have a [protocol](https://www2.healthservice.hse.ie/organisation/national-pppgs/guidelines-for-safe-effective-management-and-use-of-point-of-care-testing-in-primary-and-community-care/#:~:text=Point%20of%20care%20testing%20is,outside%20the%20conventional%20laboratory%20environment).  I don't know how onerous the maintenance is. It is difficult to answer this.  HSE should undertake quality control or reimbursement of same.  Maintenance by the manufacturer is always more expert, professional and reliable. Expertise is important here and manufacturers would be best placed. |
| --- |
