# Supplementary material for: Point-of-caRE DiagnostICs for respiraTOry tRact infectionS (PREDICTORS) study: developing guidance for using C-reactive protein point-of-care tests in the management of lower respiratory tract infections in primary care using a Delphi consensus technique
Source: BMJ Open. 2025 May 27;15(5):e101438. doi: 10.1136/bmjopen-2025-101438 (PMC12121597; doi:10.1136/bmjopen-2025-101438)
Supplement: online supplemental file 6 [file bmjopen-15-5-s006.docx]

# Supporting Information File 6: Round 2 Comments

Please note all comments are verbatim.

## Criteria that required further consideration (n = 9)

### Section 1: Using CRP POCT

1. With appropriate training for the clinical assessment of patients suspected of having a LRTI, healthcare professionals in general practice, other than GPs themselves (i.e., advanced nurse practitioners, general practice pharmacists) should be able to obtain and interpret CRP POCT results with GP oversight.

| Workload time-constraints on pharmacists and remuneration within the current community pharmacy contract would need careful consideration to make such enhanced practice feasible for the profession.  I would agree for straightforward LRTIs but I think most LRTIs need to be seen by a GP so it depends on what oversight means. Would certainly help the GP having some supporting tests.  Outside of Ireland and the UK, I would see the buy-in from GPs in Europe very hard to achieve and so, not sure how easy it would be to create and publish protocols and guidelines within this area.  This would undoubtedly alleviate a lot of the pressures on GPs, and could mirror the minor ailments scheme in practice in the UK.  Would still require some GP time.  I am uncertain as to whether our medical indemnity would cover other healthcare professionals to take on extra roles.  I do not think that care should be fragmented. Part of this will depend on the detail such as how long the NPT for CRP takes. If it is quick and can be done in consultation, that is the best place for it.  Using skilled healthcare professionals within the remits of their roles to complete tasks should ensure that all healthcare professionals including GPs have the time to complete the aspects of the test that only they can do, thus being more efficient with the limited resources available. |
| --- |

1. With appropriate training for treating patients suspected of having a LRTI, healthcare professionals in general practice other than GPs themselves (i.e., advanced nurse practitioners, general practice pharmacists) should be able to act upon CRP POCT results (i.e., prescribe antibiotic therapy when indicated following national/international antibiotic prescribing guidance or provide self-care advice to patients) with GP oversight.

| Once the condition is diagnosed, the pharmacist may be better positioned to prescribe and act upon the information. As the medicines experts their time might be better spent selecting and dispensing appropriate meds rather than diagnosing and liaising with GPs. It is a matter of time-management in the current pharmacy practice model. If the remuneration model were to change however, this may make a workable difference and enable greater patient access, similar to vaccination strategies.  See previous. many LRTI patients have comorbidity/COPD etc so GP clinical assessment needed.  I think the only way this system would work is if it is driven primarily by healthcare professionals in general practice other than GPs from start to finish of the process. If the guidelines clearly outline treatment recommendations as per guidelines, there is no reason why these trained healthcare professionals couldn't interpret the results and make appropriate recommendations with GP oversight.  With appropriate training.  With caveat as above.  I don’t think it will reduce their work. I think that the number of times the prescription will reduce work will be less than LB 1 in 4.  Role substitution has not worked well in other jurisdictions eg the UK and the USA. I do not believe that antibiotic prescribing should be liberalised. I do not think that is appropriate in the light of the urgent need to manage antimicrobial resistance.  It has been shown in evidence and anecdotally (e.g. in the UK) that non-medical prescribing of antibiotics is safe and appropriate. Would recommend that specialist training in AMS/antibiotic prescribing is important here.  Within a set criteria and when trained to complete this task, appropriate other healthcare professionals can complete this, with referral to GPs when outside the scope or parameters of the guidance. |
| --- |

1. CRP POCT should be performed within community pharmacies by community pharmacists who have received appropriate training, adhere to approved protocols, and liaise with or refer to the patient’s GP when necessary.

| As above, Once the condition is diagnosed, the pharmacist may be better positioned to prescribe and act upon the information. As the medicines experts their time might be better spent selecting and dispensing appropriate meds rather than diagnosing and liaising with GPs. It is a matter of time-management in the current pharmacy practice model.  Depends on sensitivity and specificity of test. PPVs etc Could falsely reassure someone screening a patient.  Again - this would be in line with the work from the Expert taskforce to expand the role of the pharmacist. I think community pharmacists are in a perfect position to offer this service, provided they are given adequate support structures and appropriate training is put in place.  Results not integrated onto patient chart in a seamless way. Would create additional work for GP - who would probably want to see the patient themselves if being asked to prescribe.  This fragments care and dilutes clinical governance. I also believe that liberalising antibiotic availability is a bad idea. Ireland is already the 8th highest prescriber of antibiotics in the EU. Broadening access is the opposite of what is needed for good antimicrobial stewardship.  Being accessible will ensure that this test can be used to provide care to patients and refer appropriate patients to prescribers. |
| --- |

1. With appropriate training for the clinical assessment of patients suspected of having a LRTI, community pharmacists should be able to obtain and interpret CRP POCT results, provided they liaise with or refer to the patient’s GP when necessary.

| See above.  As above.  The service is only beneficial if the pharmacist can also prescribe based on it.  There tends to be a lot more nuance to diagnosis than just one CRP test result - will work to reassure those who were clearly not too unwell anyway without CRP testing but much trickier for the other situations.  LRTI needs more than CRP.  Doctors train for years in diagnosis and have expertise in this. Pharmacists are highly expert in medication safety and drug interactions. I believe that each specialist (pharmacist/GP) should play to their strengths. I do not think that adding complexity to communication channels will be beneficial to patients. I don't agree with fragmenting clinical care. |
| --- |

1. With appropriate training for treating patients suspected of having a LRTI, community pharmacists should be able to act upon CRP POCT results (i.e., prescribe antibiotic therapy when indicated following national/international antibiotic prescribing guidance or provide self-care advice to patients), provided they liaise with or refer to the patient's GP when necessary.

| Pharmacists' time is limited in community practice and such proposals may prove unfeasible if they are not given full scope to be able to dispense the appropriate meds without having to liaise with the GP. Duplication of roles would not necessarily help the healthcare system overall.  see previous comments. GP assessment needed for LRTI. Different for URTI.  I think community pharmacies are perfectly positioned to address this patient need. More often than not, this cohort of patients present to the pharmacy with sore throat symptoms in the hope that they can get an OTC remedy. CRP POCT results would ensure patients are treated most appropriately with the best care right from the beginning of presentations of symptoms.  I do not believe that antibiotic prescribing is appropriate based on CRP only and without full clinical assessment for LRTI and ruling out other causes of symptoms.  Why not have a viral test? That excludes rather than includes.  Antibiotic resistance is a significant threat. I do not agree with liberalising access to these medications. As a nation we already prescribe too many antibiotics. As before, there are risks with fragmenting care. Communication will become increasingly complex with such a model with the associated risks of clinical error.  I would be cautious to propose the only reason for pharmacist prescribing would be to reduce pressure on GPs. Careful consideration of the impact of more antibiotic prescribers is needed. Risks include an inadvertent increase in antibiotic prescribing, antimicrobial resistance. Resource and investment into training and diagnostic tests (such as CRP) are recommended. Pharmacists should ideally have access to the patients medical history to comprehensively prescribe an antibiotic and consider all issues e.g. interactions, previous infection, recent antibiotic use etc. |
| --- |

### Section 2: The detection of bacterial LRTIs using CRP POCT and the provision of antibiotics

1. In cases where a child is presented early in the progression of symptoms (i.e., in the first 24 hours), CRP results should be interpreted carefully, with attention to the clinical context and severity of illness. In this instance, a CRP value <5 mg/L may indicate a self-limiting infection (bacterial or viral) for which antibiotics should not be prescribed.

| Depends on what interpreted carefully means - I don't follow that. CRP testing is part of an overall clinical assessment and hard to interpret alone.  As there can be a lag time in the raising of CRP I don't think it can be used as a reliable tool to rule out infection in the first 24 hours.  Doing invasive testing on children even a finger prick is a big deal and not routine.  Early in the course of illness biomarkers are of limited value and require significant safety netting.  I agree that a CRP value under 5 MAY indicate a self limiting infection. |
| --- |

1. For CRP values between 20-100 mg/L in low-risk patients (i.e., not at a higher risk of deterioration due to existing conditions or severe symptoms), prescribing of antibiotics should be avoided or delayed following national/international antibiotic prescribing guidance, though clinical judgement and patient-specific factors should be considered, with clear communication of self-care and re-referral advice to the patient.

| This seems like a step in the right direction away from antimicrobial resistance.  Within GP setting only.  Not nearly enough information.  I agree generally, although the clinical context is crucial in making this decision. |
| --- |

### Section 3: Communication strategies to increase antibiotic stewardship

1. For CRP POCT conducted in community pharmacies, patients should be provided with a detailed description of the assessment undertaken and the results obtained so they can provide these to their GP if necessary.

| Agree, full access to testing information is important for transparency and any potential follow-up.  not clear that this statement relates to LRTI or URTI.  Likely to create additional work load that is not accounted for on the patient diary in the practice.  As above this will add to GP work.  I think that these clinical assessments should be done in GP, but if they are done in a community pharmacy, it is helpful to have access to the CRP result.  With consent from the patient. |
| --- |

### Section 5: User operation of CRP POCT

1. CRP POCT providers (e.g., GPs, community pharmacists and advanced nurse practitioners) should undergo training to use the POCT device and to interpret CRP results, combined with enhanced communication skills training to effectively convey findings and implications to patients.

| Agree that this is important for all the professions.  Who will pay?  In my view the CRP POCT testing should be done in GP rather than elsewhere I order to avoid fragmentation of care, to have clearer governance structures and to avoid increasing the risk of communication failures.  Added to this is training on explaining to patients the risks associated with an antibiotic (e.g. ADRs, resistance) and the evidence to support self-management/non-antibiotic prescribing clearly. |
| --- |

## Criteria fundamentally accepted but wording slightly changed (n = 15)

### Section 1: Using CRP POCT

1. A thorough clinical assessment of the patient’s history (e.g., comorbidities, previous admissions), risk profile, and acute clinical situation should precede CRP POCT.

| Need more than that.  CRP POCT can on its own given not a complete picture of the patient's condition and the need for treatment or not so history and clinical details should be gathered |
| --- |

1. Validated, evidence-based clinical decision rules (e.g., STARWAVe and CRB-65) should be incorporated into the clinical assessment of patients suspected of having a LRTI.

| But clinical assessment may override a clinical decision rule - they are only meant to be guidance and support for decision making.  These tools also have limitations which would need to be outlined clearly. e.g. CRB-65 i some younger patients.  They are not well validated for community setting where that is the only information used. |
| --- |

1. CRP POCT should be used for patients suspected of having a LRTI only when the prescriber is uncertain about prescribing antibiotics for LRTIs following their clinical assessment of the patient.

| CRP POCT should be used more broadly to prevent inappropriate prescribing.  May be useful even if not thinking of prescribing an antibiotic  I don't personally like the tightness the word "only" puts here - I'd be much more of a hand rail as opposed to train track kind of healthcare practioner i.e. I like guidelines to allow for wiggle room  Depending on the cost associated with it, should CRP POCT be performed in all cases to remove any ambiguity around symptoms.  The test should only be done if it is like to alter management. If the clinical decision is reasonably certain, there is no benefit to doing the test  In cases of uncertainty, CRP may help to reduce inappropriate antibiotic prescribing.  CRP POCT can be an add on layer of information, if history, clinical presentation etc when assessed is very clear that it is a LRTI (or it is not a LRTI) then there should be no need to complete CRP POCT however it can be of benefit it unclear and additional information is required in the decision making process. |
| --- |

1. CRP POCT should aid appropriate prescribing of antibiotics for LRTIs by reducing diagnostic uncertainty as part of the clinical assessment.

| A test that diagnosed a virus would be much better and simpler. |
| --- |

1. Consent, either written or implied, should be obtained from the patient or the patient’s parent or legal guardian in the case of children below the age of 16 for their blood sample to be taken for CRP POCT. Additionally, where appropriate, children should provide their assent.

| Formal consent not needed in route clinical practice - is implied. |
| --- |

1. CRP POCT should be performed within general practice by general practitioners (GPs), guided by clinical judgement and current best practice guidance.

| If there is evidence of its cost effectiveness and in appropriate target patients.  I agree that is should be conducted within general practice by GPs - but I feel it should also be conducted in other settings as outlined previously.  Not necessarily if other HCP has completed appropriate training and is working within the parameters of clear guidance. |
| --- |

1. CRP POCT should be used in conjunction with a rapid viral test (e.g., SARS-Cov-2/Influenza A&B/RSV Adenovirus antigen combo rapid test) in the presence of cough and fever during an active pandemic or epidemic (such as COVID-19 or Influenza A&B). This is contingent upon the tests demonstrating appropriate sensitivity, specificity, and suitability for the context and population.

| I am not sure if there is enormous benefit to testing for RSV/adenovirus - there are no disease-specific interventions for those. Paxlovid and oseltamivir have limited benefits at best, but they may be appropriate in some situations. |
| --- |

### Section 2: The detection of bacterial LRTIs using CRP POCT and the provision of antibiotics

1. A CRP value >20 mg/L may indicate the possibility of a bacterial LRTI, though clinical judgement should be used as CRP levels can also be elevated in viral infections (although CRP elevations due to viral infections typically remain below 20 mg/L).

| Seems like strange indicator - this is more a statement of fact than an opinion/recommendation.  When a statement includes the phrase "may" it is difficult to ever disagree with it.  Details on clinical signs and symptoms to assess the clinical presentation are important in this type of scenario. If no antibiotic prescribed, clear information to the patient on safety netting is needed. What to do if particular symptoms arise. |
| --- |

1. A CRP value <20 mg/L may indicate a self-limiting infection (bacterial or viral) for which antibiotics should not be prescribed, though clinical judgement and patient-specific factors should be considered, with clear communication of self-care and re-referral advice to the patient.

| Ensure to rule out other causes e.g. heart failure in SOB, lung cancer in cough.  When a statement includes the phrase "may" it is difficult to ever disagree with it. |
| --- |

1. CRP values >100 mg/L in adults indicates the presence of a severe infection for which antibiotics should be prescribed following national/international antibiotic prescribing guidance and hospital referral considered alongside thorough clinical assessment, with attention to the clinical context, severity of illness, and potential non-bacterial causes of elevated CRP levels.

| CRP values >100 mg/L in adults indicates the presence of severe INFLAMMATION which may be due to a severe infection for which antibiotics may need to be prescribed.  This is a general comment, somewhat outside the remit of this study, but written referral from a pharmacist to a GP/hospital should become the norm along with a verbal instruction to the patient to attend their GP or local ED. |
| --- |

1. CRP values >75 mg/L in children indicates the presence of a severe infection for which antibiotics should be prescribed following national/international antibiotic prescribing guidance and hospital referral considered alongside thorough clinical assessment, with attention to the clinical context, severity of illness, and potential non-bacterial causes of elevated CRP levels.

| CRP values >75 mg/L in children indicates the presence of severe INFLAMMATION which may be caused by a severe infection.  These children are at high risk of infection.  I think that further consultation with paed emergency medicine/respiratory physicians should be sought here. |
| --- |

1. For CRP values between 20-100 mg/L in patients with a higher risk of deterioration (i.e., due to existing conditions or severe symptoms), antibiotic prescribing following national/international antibiotic prescribing guidance should be considered, with attention to the clinical context, severity of illness, and potential non-bacterial causes of elevated CRP levels.

| When a statement includes the phrase "should be considered" it is difficult to ever disagree with it. |
| --- |

### Section 4: Features and performance of the CRP POCT device

1. Results should be stored on the CRP POCT device or in the patient’s record, with consideration for ease of use and integration into clinical workflow, while ensuring patient privacy and data security.

| Cloud storage may be better and more secure.  Good clinical records important.  Results must be stored in a patient record and not just on the CRP device.  Uncertain on the security of the POCT device, e.g. can this be used as an indelible patient record the same as an EPR or would it be at risk of losing patients' data. Can it be connected to the pharmacy patient record instead?  Results should be stored as according to the legal requirements for storing of clinical records. The device may not be the most appropriate place to store if there is a risk of breaches of data confidentiality. But the results should be backed up in the patients records for review in the future. |
| --- |

1. CRP POCT results should be automatically transferred from the device to patients’ records where feasible and efficient, leveraging fully integrated electronic healthcare records or easily scannable formats, to streamline documentation and ensure accurate record-keeping.

| The devil is in the detail here - there is a risk of disseminating medicolegal risk. It depends on the specifics.  This would be a better way for storing records. |
| --- |

### Section 5: User operation of CRP POCT

1. Maintenance, calibration and quality control will be required for the CRP POCT device as per manufacturer recommendations, with support and oversight provided by CRP POCT device supplier (e.g., device manufacturer).

| Maintenance, calibration and quality control may be better performed by the manufacturers as the experts, rather than just having oversight. |
| --- |
