# Supplementary material for: Point-of-caRE DiagnostICs for respiraTOry tRact infectionS (PREDICTORS) study: developing guidance for using C-reactive protein point-of-care tests in the management of lower respiratory tract infections in primary care using a Delphi consensus technique
Source: BMJ Open. 2025 May 27;15(5):e101438. doi: 10.1136/bmjopen-2025-101438 (PMC12121597; doi:10.1136/bmjopen-2025-101438)
Supplement: online supplemental file 7 [file bmjopen-15-5-s007.docx]

**Supporting Information Table 1: Delphi panel responses from Round 1**

| **Criterion** | **Delphi Panel Member** | | | | | | | | | | | | | | | | | | |
| --- | --- | --- | --- | --- | --- | --- | --- | --- | --- | --- | --- | --- | --- | --- | --- | --- | --- | --- | --- |
|  | **1** | **2** | **3** | **4** | **5** | **6** | **7** | **8** | **9** | **10** | **11** | **12** | **13** | **14** | **15** | **16** | **17** | **18** | **19** |
| 1 | SA | D | D | U | D | U | SD | SD | D | U | A | A | D | A | SA | D | A | D | A |
|  | 5 | 2 | 2 | 3 | 2 | 3 | 1 | 1 | 2 | 3 | 4 | 4 | 2 | 4 | 5 | 2 | 4 | 2 | 4 |
| 2 | A | A | A | A | A | A | U | SA | A | A | U | A | A | D | D | SA | A | A | SA |
|  | 4 | 4 | 4 | 4 | 4 | 4 | 3 | 5 | 4 | 4 | 3 | 4 | 4 | 2 | 2 | 5 | 4 | 4 | 5 |
| 3 | A | A | A | U | A | SA | SA | D | U | A | A | A | A | A | SA | SA | A | D | SA |
|  | 4 | 4 | 4 | 3 | 4 | 5 | 5 | 2 | 3 | 4 | 4 | 4 | 4 | 4 | 5 | 5 | 4 | 2 | 5 |
| 4 | A | SA | A | A | SA | A | SA | SA | SA | SD | SA | A | A | A | SA | SA | A | SA | A |
|  | 4 | 5 | 4 | 4 | 5 | 4 | 5 | 5 | 5 | 1 | 5 | 4 | 4 | 4 | 5 | 5 | 4 | 5 | 4 |
| 5 | A | SA | A | A | A | A | SA | SA | A | A | SA | A | A | SA | SA | SA | U | A | A |
|  | 4 | 5 | 4 | 4 | 4 | 4 | 5 | 5 | 4 | 4 | 5 | 4 | 4 | 5 | 5 | 5 | 3 | 4 | 4 |
| 6 | SA | SA | SA | SA | SA | SA | SA | SA | SA | SD | SA | SA | A | SA | SA | SA | SA | SA | SA |
|  | 5 | 5 | 5 | 5 | 5 | 5 | 5 | 5 | 5 | 1 | 5 | 5 | 4 | 5 | 5 | 5 | 5 | 5 | 5 |
| 7 | A | SA | SA | A | SD | A | SD | SA | SA | A | A | A | A | A | SA | SA | A | SA | A |
|  | 4 | 5 | 5 | 4 | 1 | 4 | 1 | 5 | 5 | 4 | 4 | 4 | 4 | 4 | 5 | 5 | 4 | 5 | 4 |
| 8 | A | SA | SA | A | A | A | SA | U | A | A | A | U | A | SA | SA | U | A | A | SA |
|  | 4 | 5 | 5 | 4 | 4 | 4 | 5 | 3 | 4 | 4 | 4 | 3 | 4 | 5 | 5 | 3 | 4 | 4 | 5 |
| 9 | SA | SA | A | A | A | A | SD | SD | SA | D | SA | U | A | U | SA | A | D | U | A |
|  | 5 | 5 | 4 | 4 | 4 | 4 | 1 | 1 | 5 | 2 | 5 | 3 | 4 | 3 | 5 | 4 | 2 | 3 | 4 |
| 10 | SA | A | A | A | U | A | SD | SD | SA | D | A | N/A | U | A | SA | SA | D | U | A |
|  | 5 | 4 | 4 | 4 | 3 | 4 | 1 | 1 | 5 | 2 | 4 | N/A | 3 | 4 | 5 | 5 | 2 | 3 | 4 |
| 11 | SD | A | SA | U | SA | A | SA | SA | D | A | U | N/A | A | D | SA | U | A | A | A |
|  | 1 | 4 | 5 | 3 | 5 | 4 | 5 | 5 | 2 | 4 | 3 | N/A | 4 | 2 | 5 | 3 | 4 | 4 | 4 |
| 12 | N/A | U | U | U | U | A | U | SD | A | U | SA | N/A | U | A | A | SA | D | U | A |
|  | N/A | 3 | 3 | 3 | 3 | 4 | 3 | 1 | 4 | 3 | 5 | N/A | 3 | 4 | 4 | 5 | 2 | 3 | 4 |
| 13 | U | U | A | U | U | A | U | SD | A | U | SA | N/A | U | A | A | SA | D | A | A |
|  | 3 | 3 | 4 | 3 | 3 | 4 | 3 | 1 | 4 | 3 | 5 | N/A | 3 | 4 | 4 | 5 | 2 | 4 | 4 |
| 14 | U | U | A | U | U | U | U | SD | A | D | SA | A | D | A | A | SA | D | A | A |
|  | 3 | 3 | 4 | 3 | 3 | 3 | 3 | 1 | 4 | 2 | 5 | 4 | 2 | 4 | 4 | 5 | 2 | 4 | 4 |
| 15 | SA | A | A | A | SA | U | U | D | A | A | U | U | D | D | SA | U | A | A | A |
|  | 5 | 4 | 4 | 4 | 5 | 3 | 3 | 2 | 4 | 4 | 3 | 3 | 2 | 2 | 5 | 3 | 4 | 4 | 4 |
| 16 | A | SD | SA | U | U | A | U | SD | A | U | D | A | U | D | U | A | D | A | U |
|  | 4 | 1 | 5 | 3 | 3 | 4 | 3 | 1 | 4 | 3 | 2 | 4 | 3 | 2 | 3 | 4 | 2 | 4 | 3 |
| 17 | U | SD | U | A | D | A | U | SD | D | SD | A | D | D | D | A | U | A | A | D |
|  | 3 | 1 | 3 | 4 | 2 | 4 | 3 | 1 | 2 | 1 | 4 | 2 | 2 | 2 | 4 | 3 | 4 | 4 | 2 |
| 18 | A | SA | SA | SA | SA | SA | SA | SA | SA | A | SA | N/A | A | SA | SA | SA | A | SA | SA |
|  | 4 | 5 | 5 | 5 | 5 | 5 | 5 | 5 | 5 | 4 | 5 | N/A | 4 | 5 | 5 | 5 | 4 | 5 | 5 |
| 19 | SA | SA | A | A | SA | A | U | U | A | SA | SA | A | U | A | SA | SA | A | A | A |
|  | 5 | 5 | 4 | 4 | 5 | 4 | 3 | 3 | 4 | 5 | 5 | 4 | 3 | 4 | 5 | 5 | 4 | 4 | 4 |
| 20 | SA | SA | SA | A | SA | U | A | A | SA | SA | SA | A | A | SA | SA | SA | SA | A | SA |
|  | 5 | 5 | 5 | 4 | 5 | 3 | 4 | 4 | 5 | 5 | 5 | 4 | 4 | 5 | 5 | 5 | 5 | 4 | 5 |
| 21 | SA | SA | SA | A | SA | U | A | A | SA | SA | SA | A | A | SA | SA | SA | SA | A | SA |
|  | 5 | 5 | 5 | 4 | 5 | 3 | 4 | 4 | 5 | 5 | 5 | 4 | 4 | 5 | 5 | 5 | 5 | 4 | 5 |
| 22 | SA | U | SA | A | U | A | A | A | SA | U | SA | A | A | SA | SA | SA | SA | A | SA |
|  | 5 | 3 | 5 | 4 | 3 | 4 | 4 | 4 | 5 | 3 | 5 | 4 | 4 | 5 | 5 | 5 | 5 | 4 | 5 |
| 23 | A | SA | SA | SA | SA | A | A | SD | A | SA | SA | A | A | SA | SA | A | A | A | A |
|  | 4 | 5 | 5 | 5 | 5 | 4 | 4 | 1 | 4 | 5 | 5 | 4 | 4 | 5 | 5 | 4 | 4 | 4 | 4 |
| 24 | A | A | A | U | A | A | D | U | A | SA | SA | A | A | A | A | U | A | A | A |
|  | 4 | 4 | 4 | 3 | 4 | 4 | 2 | 3 | 4 | 5 | 5 | 4 | 4 | 4 | 4 | 3 | 4 | 4 | 4 |
| 25 | A | A | A | A | SA | U | A | SA | U | A | SA | A | U | A | A | A | A | A | SA |
|  | 4 | 4 | 4 | 4 | 5 | 3 | 4 | 5 | 3 | 4 | 5 | 4 | 3 | 4 | 4 | 4 | 4 | 4 | 5 |
| 26 | A | A | A | U | U | A | A | U | A | U | U | A | U | SA | A | U | U | U | U |
|  | 4 | 4 | 4 | 3 | 3 | 4 | 4 | 3 | 4 | 3 | 3 | 4 | 3 | 5 | 4 | 3 | 3 | 3 | 3 |
| 27 | A | SA | SA | A | A | A | D | A | A | SA | SA | A | SA | SA | SA | A | A | A | SA |
|  | 4 | 5 | 5 | 4 | 4 | 4 | 2 | 4 | 4 | 5 | 5 | 4 | 5 | 5 | 5 | 4 | 4 | 4 | 5 |
| 28 | A | A | SA | U | SA | SA | U | A | A | SA | A | A | A | SA | SA | A | A | A | SA |
|  | 4 | 4 | 5 | 3 | 5 | 5 | 3 | 4 | 4 | 5 | 4 | 4 | 4 | 5 | 5 | 4 | 4 | 4 | 5 |
| 29 | A | A | D | A | A | D | U | A | A | A | A | A | U | A | SA | U | A | A | A |
|  | 4 | 4 | 2 | 4 | 4 | 2 | 3 | 4 | 4 | 4 | 4 | 4 | 3 | 4 | 5 | 3 | 4 | 4 | 4 |
| 30 | A | A | SA | A | SA | SA | A | SA | A | SA | A | A | A | SA | SA | A | A | A | A |
|  | 4 | 4 | 5 | 4 | 5 | 5 | 4 | 5 | 4 | 5 | 4 | 4 | 4 | 5 | 5 | 4 | 4 | 4 | 4 |
| 31 | SA | SA | SA | SA | SA | A | SA | A | SA | SA | SA | A | SA | A | SA | SA | A | SA | SA |
|  | 5 | 5 | 5 | 5 | 5 | 4 | 5 | 4 | 5 | 5 | 5 | 4 | 5 | 4 | 5 | 5 | 4 | 5 | 5 |
| 32 | SA | SA | A | A | A | A | SA | A | SA | SA | SA | A | A | A | SA | SA | A | A | SA |
|  | 5 | 5 | 4 | 4 | 4 | 4 | 5 | 4 | 5 | 5 | 5 | 4 | 4 | 4 | 5 | 5 | 4 | 4 | 5 |
| 33 | SA | A | D | A | U | A | A | SD | A | SD | SA | A | U | A | SA | A | D | U | D |
|  | 5 | 4 | 2 | 4 | 3 | 4 | 4 | 1 | 4 | 1 | 5 | 4 | 3 | 4 | 5 | 4 | 2 | 3 | 2 |
| 34 | SA | SA | D | U | SA | SA | SA | D | A | SD | SA | A | A | A | SA | A | A | U | SA |
|  | 5 | 5 | 2 | 3 | 5 | 5 | 5 | 2 | 4 | 1 | 5 | 4 | 4 | 4 | 5 | 4 | 4 | 3 | 5 |
| 35 | SA | SA | SA | U | SA | SA | A | SA | SA | A | SA | A | A | A | SA | A | A | A | SA |
|  | 5 | 5 | 5 | 3 | 5 | 5 | 4 | 5 | 5 | 4 | 5 | 4 | 4 | 4 | 5 | 4 | 4 | 4 | 5 |
| 36 | SA | SA | D | A | SA | A | SA | SA | SA | SA | SA | A | SA | A | SA | A | A | A | SA |
|  | 5 | 5 | 2 | 4 | 5 | 4 | 5 | 5 | 5 | 5 | 5 | 4 | 5 | 4 | 5 | 4 | 4 | 4 | 5 |
| 37 | SA | SA | D | A | SA | A | SA | SA | SA | SA | SA | A | SA | A | SA | A | A | A | SA |
|  | 5 | 5 | 2 | 4 | 5 | 4 | 5 | 5 | 5 | 5 | 5 | 4 | 5 | 4 | 5 | 4 | 4 | 4 | 5 |
| 38 | SA | SA | A | A | SA | A | SA | SA | SA | A | SA | A | SA | A | SA | A | A | A | SA |
|  | 5 | 5 | 4 | 4 | 5 | 4 | 5 | 5 | 5 | 4 | 5 | 4 | 5 | 4 | 5 | 4 | 4 | 4 | 5 |
| 39 | SA | SA | A | A | SA | A | SA | SA | SA | U | SA | A | SA | A | D | A | A | A | SA |
|  | 5 | 5 | 4 | 4 | 5 | 4 | 5 | 5 | 5 | 3 | 5 | 4 | 5 | 4 | 2 | 4 | 4 | 4 | 5 |
| 40 | SA | SA | A | A | SA | U | SA | SA | SA | SA | SA | A | A | A | SA | A | SA | A | SA |
|  | 5 | 5 | 4 | 4 | 5 | 3 | 5 | 5 | 5 | 5 | 5 | 4 | 4 | 4 | 5 | 4 | 5 | 4 | 5 |
| 41 | SA | SA | A | A | SA | SA | SA | SA | SA | SA | SA | A | SA | A | SA | A | SA | A | SA |
|  | 5 | 5 | 4 | 4 | 5 | 5 | 5 | 5 | 5 | 5 | 5 | 4 | 5 | 4 | 5 | 4 | 5 | 4 | 5 |
| 42 | SA | SA | A | A | SA | A | SA | SA | A | SA | SA | A | A | A | SA | A | SA | A | SA |
|  | 5 | 5 | 4 | 4 | 5 | 4 | 5 | 5 | 4 | 5 | 5 | 4 | 4 | 4 | 5 | 4 | 5 | 4 | 5 |
| 43 | SA | A | A | A | SA | D | U | U | A | SA | SA | A | A | D | SA | A | SA | A | SA |
|  | 5 | 4 | 4 | 4 | 5 | 2 | 3 | 3 | 4 | 5 | 5 | 4 | 4 | 2 | 5 | 4 | 5 | 4 | 5 |
| 44 | SA | SA | A | A | SA | SA | A | SA | SA | D | SA | A | A | SA | SA | A | U | A | SA |
|  | 5 | 5 | 4 | 4 | 5 | 5 | 4 | 5 | 5 | 2 | 5 | 4 | 4 | 5 | 5 | 4 | 3 | 4 | 5 |
| 45 | SA | SA | A | A | U | D | SA | SA | SA | A | SA | A | SA | U | SA | A | SA | D | D |
|  | 5 | 5 | 4 | 4 | 3 | 2 | 5 | 5 | 5 | 4 | 5 | 4 | 5 | 3 | 5 | 4 | 5 | 2 | 2 |
| 46 | SA | SA | A | A | A | A | SA | SA | SA | SA | SA | A | SA | SA | SA | A | SA | D | A |
|  | 5 | 5 | 4 | 4 | 4 | 4 | 5 | 5 | 5 | 5 | 5 | 4 | 5 | 5 | 5 | 4 | 5 | 2 | 4 |
| 47 | SA | SA | A | A | SA | SA | SA | SA | SA | A | SA | A | SA | SA | SA | SA | SA | A | SA |
|  | 5 | 5 | 4 | 4 | 5 | 5 | 5 | 5 | 5 | 4 | 5 | 4 | 5 | 5 | 5 | 5 | 5 | 4 | 5 |
| 48 | SA | SA | A | A | SA | SA | SA | SA | SA | A | SA | A | SA | D | SA | SA | SA | SA | SA |
|  | 5 | 5 | 4 | 4 | 5 | 5 | 5 | 5 | 5 | 4 | 5 | 4 | 5 | 2 | 5 | 5 | 5 | 5 | 5 |
| 49 | U | SA | D | A | A | SA | SD | U | SA | N/A | A | A | SA | SD | SA | SA | A | SA | SA |
|  | 3 | 5 | 2 | 4 | 4 | 5 | 1 | 3 | 5 | N/A | 4 | 4 | 5 | 1 | 5 | 5 | 4 | 5 | 5 |

Abbreviations: *SA* strongly agree, *A* agree, *U* uncertain, *D* disagree, *SD* strongly disagree
