# Supplementary material for: Point-of-caRE DiagnostICs for respiraTOry tRact infectionS (PREDICTORS) study: developing guidance for using C-reactive protein point-of-care tests in the management of lower respiratory tract infections in primary care using a Delphi consensus technique
Source: BMJ Open. 2025 May 27;15(5):e101438. doi: 10.1136/bmjopen-2025-101438 (PMC12121597; doi:10.1136/bmjopen-2025-101438)
Supplement: online supplemental file 8 [file bmjopen-15-5-s008.docx]

**Supporting Information Table 2: Delphi panel responses from Round 2**

| **Criterion** | **Delphi Panel Member** | | | | | | | | | | | | | | | |
| --- | --- | --- | --- | --- | --- | --- | --- | --- | --- | --- | --- | --- | --- | --- | --- | --- |
|  | **1** | **2** | **3** | **4** | **5** | **8** | **9** | **10** | **11** | **12** | **13** | **14** | **15** | **16** | **18** | **19** |
| 1 | SA | A | U | A | SA | SD | SA | A | SA | A | U | U | SA | SA | U | A |
|  | 5 | 4 | 3 | 4 | 5 | 1 | 5 | 4 | 5 | 4 | 3 | 3 | 5 | 5 | 3 | 4 |
| 2 | SA | A | A | A | U | SD | SA | A | SA | U | U | A | SA | SA | U | A |
|  | 5 | 4 | 4 | 4 | 3 | 1 | 5 | 4 | 5 | 3 | 3 | 4 | 5 | 5 | 3 | 4 |
| 3 | SA | A | A | U | SA | SD | SA | U | SA | U | D | A | SA | SA | U | A |
|  | 5 | 4 | 4 | 3 | 5 | 1 | 5 | 3 | 5 | 3 | 2 | 4 | 5 | 5 | 3 | 4 |
| 4 | SA | U | D | U | A | SD | A | D | SA | U | D | U | SA | SA | U | A |
|  | 5 | 3 | 2 | 3 | 4 | 1 | 4 | 2 | 5 | 3 | 2 | 3 | 5 | 5 | 3 | 4 |
| 5 | SA | A | A | U | U | SD | A | D | SA | U | D | U | SA | SA | U | U |
|  | 5 | 4 | 4 | 3 | 3 | 1 | 4 | 2 | 5 | 3 | 2 | 3 | 5 | 5 | 3 | 3 |
| 6 | SA | U | A | D | SA | SA | A | A | SA | U | U | A | A | A | U | A |
|  | 5 | 3 | 4 | 2 | 5 | 5 | 4 | 4 | 5 | 3 | 3 | 4 | 4 | 4 | 3 | 4 |
| 7 | SA | SA | A | A | SA | A | A | A | SA | U | A | A | SA | A | U | A |
|  | 5 | 5 | 4 | 4 | 5 | 4 | 4 | 4 | 5 | 3 | 4 | 4 | 5 | 4 | 3 | 4 |
| 8 | SA | A | A | A | SA | A | SA | A | SA | D | U | A | SA | A | U | SA |
|  | 5 | 4 | 4 | 4 | 5 | 4 | 5 | 4 | 5 | 2 | 3 | 4 | 5 | 4 | 3 | 5 |
| 9 | SA | SA | SA | A | SA | D | SA | A | SA | D | A | SA | SA | SA | U | SA |
|  | 5 | 5 | 5 | 4 | 5 | 2 | 5 | 4 | 5 | 2 | 4 | 5 | 5 | 5 | 3 | 5 |
| 10 | SA | SA | SA | SA | SA | SA | A | SA | SA | D | SA | SA | SA | SA | SA | SA |
|  | 5 | 5 | 5 | 5 | 5 | 5 | 4 | 5 | 5 | 2 | 5 | 5 | 5 | 5 | 5 | 5 |
| 11 | A | SA | SA | SA | A | SA | SA | U | SA | D | A | A | A | SA | SA | SA |
|  | 4 | 5 | 5 | 5 | 4 | 5 | 5 | 3 | 5 | 2 | 4 | 4 | 4 | 5 | 5 | 5 |
| 12 | SA | A | SA | SA | A | SA | U | SA | D | U | D | D | U | SA | A | A |
|  | 5 | 4 | 5 | 5 | 4 | 5 | 3 | 5 | 2 | 3 | 2 | 2 | 3 | 5 | 4 | 4 |
| 13 | SA | SA | SA | A | A | A | A | SA | SA | U | A | SA | SA | A | A | A |
|  | 5 | 5 | 5 | 4 | 4 | 4 | 4 | 5 | 5 | 3 | 4 | 5 | 5 | 4 | 4 | 4 |
| 14 | SA | A | SA | A | SA | SA | SA | SA | SA | U | D | SA | SA | SA | SA | SA |
|  | 5 | 4 | 5 | 4 | 5 | 5 | 5 | 5 | 5 | 3 | 2 | 5 | 5 | 5 | 5 | 5 |
| 15 | SA | SA | SA | A | A | SA | D | SA | SA | SA | A | SA | A | D | A | SA |
|  | 5 | 5 | 5 | 4 | 4 | 5 | 2 | 5 | 5 | 5 | 4 | 5 | 4 | 2 | 4 | 5 |
| 16 | SA | SA | U | U | SA | U | A | SA | SA | SA | A | A | SA | A | A | SA |
|  | 5 | 5 | 3 | 3 | 5 | 3 | 4 | 5 | 5 | 5 | 4 | 4 | 5 | 4 | 4 | 5 |
| 17 | SA | A | SA | A | A | SA | A | A | SA | A | A | A | A | A | A | A |
|  | 5 | 4 | 5 | 4 | 4 | 5 | 4 | 4 | 5 | 4 | 4 | 4 | 4 | 4 | 4 | 4 |
| 18 | SA | A | SA | A | A | SA | A | A | SA | A | A | SA | A | U | A | A |
|  | 5 | 4 | 5 | 4 | 4 | 5 | 4 | 4 | 5 | 4 | 4 | 5 | 4 | 3 | 4 | 4 |
| 19 | SA | U | SA | SA | SA | SA | A | SA | SA | SA | A | SA | SA | A | A | SA |
|  | 5 | 3 | 5 | 5 | 5 | 5 | 4 | 5 | 5 | 5 | 4 | 5 | 5 | 4 | 4 | 5 |
| 20 | SA | U | SA | SA | SA | SA | U | SA | SA | U | A | SA | SA | A | A | SA |
|  | 5 | 3 | 5 | 5 | 5 | 5 | 3 | 5 | 5 | 3 | 4 | 5 | 5 | 4 | 4 | 5 |
| 21 | SA | A | SA | A | A | SA | A | SA | SA | U | A | A | SA | A | U | U |
|  | 5 | 4 | 5 | 4 | 4 | 5 | 4 | 5 | 5 | 3 | 4 | 4 | 5 | 4 | 3 | 3 |
| 22 | SA | A | D | A | SA | SA | U | SA | SA | A | SA | A | SA | A | SA | SA |
|  | 5 | 4 | 2 | 4 | 5 | 5 | 3 | 5 | 5 | 4 | 5 | 4 | 5 | 4 | 5 | 5 |
| 23 | SA | A | SA | SA | SA | A | SA | SA | SA | SA | A | A | SA | SA | SA | SA |
|  | 5 | 4 | 5 | 5 | 5 | 4 | 5 | 5 | 5 | 5 | 4 | 4 | 5 | 5 | 5 | 5 |
| 24 | SA | SA | SA | A | SA | SA | SA | SA | SA | SA | SA | U | SA | SA | SA | SA |
|  | 5 | 5 | 5 | 4 | 5 | 5 | 5 | 5 | 5 | 5 | 5 | 3 | 5 | 5 | 5 | 5 |

Abbreviations: *SA* strongly agree, *A* agree, *U* uncertain, *D* disagree, *SD* strongly disagree
