# Supplementary material for: Point-of-caRE DiagnostICs for respiraTOry tRact infectionS (PREDICTORS) study: developing guidance for using C-reactive protein point-of-care tests in the management of lower respiratory tract infections in primary care using a Delphi consensus technique
Source: BMJ Open. 2025 May 27;15(5):e101438. doi: 10.1136/bmjopen-2025-101438 (PMC12121597; doi:10.1136/bmjopen-2025-101438)
Supplement: online supplemental file 9 [file bmjopen-15-5-s009.docx]

**Supporting Information Table 3: Delphi panel responses from Round 3**

| **Criterion** |  | | | | | | | | | |
| --- | --- | --- | --- | --- | --- | --- | --- | --- | --- | --- |
|  | **1** | **2** | **3** | **4** | **5** | **8** | **9** | **10** | **11** | **12** |
| 1 | A | A | A | A | SA | A | A | A | A | A |
|  | 4 | 4 | 4 | 4 | 5 | 4 | 4 | 4 | 4 | 4 |
| 2 | A | A | SA | A | SA | U | SA | A | A | SA |
|  | 4 | 4 | 5 | 4 | 5 | 3 | 5 | 4 | 4 | 5 |
| 3 | A | A | SA | A | SA | U | A | A | A | SA |
|  | 4 | 4 | 5 | 4 | 5 | 3 | 4 | 4 | 4 | 5 |
| 4 | U | D | SA | U | SA | SD | A | A | U | A |
|  | 3 | 2 | 5 | 3 | 5 | 1 | 4 | 4 | 3 | 4 |
| 5 | D | SD | SA | U | SA | SD | A | U | U | A |
|  | 2 | 1 | 5 | 3 | 5 | 1 | 4 | 3 | 3 | 4 |
| 6 | U | SD | SA | U | SA | SD | A | U | U | A |
|  | 3 | 1 | 5 | 3 | 5 | 1 | 4 | 3 | 3 | 4 |
| 7 | SA | SA | A | U | SA | SA | A | A | A | U |
|  | 5 | 5 | 4 | 3 | 5 | 5 | 4 | 4 | 4 | 3 |

Abbreviations: *SA* strongly agree, *A* agree, *U* uncertain, *D* disagree, *SD* strongly disagree
