# Supplementary material for: Point-of-caRE DiagnostICs for respiraTOry tRact infectionS (PREDICTORS) study: developing guidance for using C-reactive protein point-of-care tests in the management of lower respiratory tract infections in primary care using a Delphi consensus technique
Source: BMJ Open. 2025 May 27;15(5):e101438. doi: 10.1136/bmjopen-2025-101438 (PMC12121597; doi:10.1136/bmjopen-2025-101438)
Supplement: Supplementary file 10 [file bmjopen-2025-101438.reviewer_comments.pdf]

PEER REVIEW HISTORY

BMJ Open publishes all reviews undertaken for accepted manuscripts. Reviewers are asked to complete a checklist review form and are provided with free text boxes to elaborate on their assessment. These free text comments are reproduced below.

ARTICLE DETAILS

Title (Provisional)

The Point-of-caRE DiagnostICs for respiraTOry tRact infectionS (PREDICTORS) Study: Developing guidance for using C-reactive protein point-of-care tests in the management of lower respiratory tract infections in primary care using a Delphi consensus technique

Authors

O'Shea, Joseph; Hughes, Carmel; Molloy, Gerard J.; Cadogan, Cathal; Vellinga, Akke; Fahey, Tom; Ryan, Cristín

---

VERSION 1 - REVIEW

**Reviewer**	**1**
**Name**	**Dewi, Ayu Putu Madri**
**Affiliation**	**Amsterdam UMC Location AMC, Epidemiology and Data Science**
**Date**	**14-Mar-2025**
**COI**	**None**

I have some feedback related to open science practices. Please find attached the feedback details.

Manuscript Title: The Point-of-caRE DiagnostICs for respiraTOry tRact infectionS (PREDICTORS) Study: Developing guidance for using C-reactive protein point-of-care tests in the management of lower respiratory tract infections in primary care using a Delphi consensus technique

Dear authors,

I am an Open Science researcher, who has checked your manuscript for its adherence to a number of Open Science practices.

Below you find the checklist that I used to assess your manuscript for adherence to a number of these practices.

Based on this assessment, I would suggest the following refinements/additions:

1. *Availability of a protocol: Please provide a (link or reference to the) the protocol of the complete study.*
2. *Pre-registration: Please provide information on whether and where the study was pre-registered.*
3. *Open Materials: Authors state "All data relevant to the study are included in the article or uploaded as supplementary information.". I think that is indeed the case, so this is okay.*
4. *Open Data: Authors state "All data relevant to the study are included in the article or uploaded as supplementary information.". I think that is indeed the case, so this is okay.*
5. *Open Code: If applicable, then please provide (a link to) the code used for the analyses in this study.*
6. *Citations: Please provide citations for your materials/data/codes; where applicable. E.g. if you used coding software for the written statements.*
7. *Software and tools used are described: Please explicitly mention whether and which software and tools were used in your study, including versions and references.*
8. *Open access publication: OK.*
9. *Reporting checklist provided: Please provide the correct completed reporting checklist (see e.g DELPHISTAR or CREDES for Delphi studies at https://www.equator-network.org/reporting-guidelines/).*
10. *Preprint: Please state explicitly whether and where the preprint of this study can be found.*

**Reviewer**	**2**
**Name**	**Melbye, Hasse**
**Affiliation**	**University of Tromsø, Institute of Community Medicine**
**Date**	**24-Mar-2025**
**COI**	**I have been a member of the ENASPOC team, financed by**
**Abbott**	

This is a well performed study on how to recommend use of the CRP rapid-test based on a Delphi process. I have only a few minor concerns. First, no experienced users of the test took part in the Delphi panels. This is mentioned in the discussion as a limitation. It is kind of strange to carry out the study without including any user, and this limitation needs to be elaborated. The other concern is related to a main controversy on when to apply the test. In the discussion you refer to this statement: "The latter report recommends that "no antibiotic should be prescribed without a test" to ensure antibiotics are only used when truly

necessary.(55)" The logic of this sentence can be questioned, but more important. it is in conflict with one of the recommendations: "CRP POCT should be used for patients suspected of having a LRTI only when the prescriber is uncertain about prescribing antibiotics for LRTIs following their clinical

assessment of the patient." The use of the test could, as an alternative, also be recommended when the care provider (GP) thinks an antibiotic is needed, as recommended in the reference. Such use of the test could make the GP change her mind, and not treat "as usual". The conflict between the reference (55) and the recommendation should be discussed.

**Reviewer**	**3**
**Name**	**Saha, Sajal**
**Affiliation**	**Monash University, Australia, General Practice**
**Date**	**12-Apr-2025**
**COI**	**None**

The work has developed the best practice guidance for using CRP POCT in the management of lower respiratory tract infections using a delphi consensus method.

The study has chosen a robust methodology and reported the results concisely. The Use of ASSURED Criteria is commendable.

However, a few areas could be improved.

1. The rationale of the sample size for delphi pannel can be explained further? Authors may follow this work. "Manyara AM, Purvis A, Ciani O, Collins GS, Taylor RS. Sample size in multistakeholder Delphi surveys: at what minimum sample size do replicability of results stabilize?. Journal of Clinical Epidemiology. 2024 Oct 1;174:111485."

2. The reliability of the responses in each round and overall could be measured if within the scope

3. The key limitation of the paper is no involvement of GPs and community pharmacists who have used CRP POCT. The consensus statements could have been different if they were included. Authors can explain why there was no involvement of those GPs/community pharmacists?

Authors have reported well the limitations including attrition bias.

VERSION 1 - AUTHOR RESPONSE

Reviewer 1: Ms. Ayu Putu Madri Dewi

- Comment: I have some feedback related to open science practices. Please find attached the feedback details.
- Response: We thank Reviewer 1 for their valuable feedback regarding open science practices. We have carefully reviewed the open science checklist and updated the manuscript to clarify our adherence to relevant practices. We have added the following statement to the Data Availability Statement section to reflect this:
- Page 17: “All data generated or analysed during this study are included in this published article and its supplementary information files. Further details on the broader study objectives and work packages can be found in the published PREDICTORS study protocol.(37) The study was not pre-registered on an open platform. No statistical code was used in the analysis and all descriptive analysis was conducted using Microsoft Excel (2024). A completed DELPHI-STAR reporting checklist has been submitted as supplementary materials. This study has not been posted as a preprint.”

Reviewer 2: Dr. Hasse Melbye

- Comment 1: I have only a few minor concerns. First, no experienced users of the test took part in the Delphi panels. This is mentioned in the discussion as a limitation. It is kind of strange to carry out the study without including any user, and this limitation needs to be elaborated.
- Response 1: We thank Reviewer 2 for this insightful comment. The selection of panellists without prior CRP POCT experience was a conscious decision aligned with the objectives of this study. Our focus was on engaging healthcare professionals working within Irish primary care, as they represent the intended end users of CRP POCT once implemented in this setting. Since CRP POCT is not currently in routine use in Irish primary care, it was not feasible to recruit experienced users locally. However, we believe that these clinicians, with their deep understanding of the Irish healthcare context, patient pathways, and operational challenges, were best placed to inform what would be important for effective adoption and use of CRP POCT in their practice. That said, we recognise that input from experienced users could offer additional practical insights, particularly regarding device handling and workflow integration. We have therefore expanded the Strengths and Limitations section to acknowledge this and to recommend that future validation work includes perspectives from healthcare professionals with hands-on experience in settings where CRP POCT is already established. The revised text now reads:
- Page 13: “It is also important to acknowledge that none of the panellists had prior experience using CRP POCT in practice, as the technology is not yet widely available in Irish primary care (Table 2). This likely influenced the perspectives captured and may have resulted in different results than if experienced users had participated. Future research should seek to validate and refine these guidance statements in real-world clinical settings, including input from healthcare professionals with hands-on experience using CRP POCT.”

- Comment 2: The other concern is related to a main controversy on when to apply the test. In the discussion you refer to this statement: "The latter report recommends that "no

antibiotic should be prescribed without a test" to ensure antibiotics are only used when truly necessary.(55)" The logic of this sentence can be questioned, but more important. it is in conflict with one of the recommendations: "CRP POCT should be used for patients suspected of having a LRTI only when the prescriber is uncertain about prescribing antibiotics for LRTIs following their clinical assessment of the patient." The use of the test could, as an alternative, also be recommended when the care provider (GP) thinks an antibiotic is needed, as recommended in the reference. Such use of the test could make the GP change her mind, and not treat "as usual". The conflict between the reference (55) and the recommendation should be discussed.
 Response 2: Thank you for highlighting this important nuance. We have revised the Discussion section to clarify the relationship between our guidance statements and international recommendations, specifically addressing the intent of the O'Neill Report (as described below) and explaining our more targeted approach to CRP POCT use. The updated text now reads:
 Page 15: "A potential tension exists between the guidance statements developed in this study and international recommendations such as those made in the O'Neill Review on Antimicrobial Resistance, which called for high-income countries to make it "mandatory that by 2020 the prescription of antibiotics will need to be informed by data and testing technology wherever it is available."(60) This recommendation reflects a push for widespread implementation of diagnostic testing to guide antibiotic use. In contrast, our Delphi panel reached consensus that CRP POCT should be used specifically when the prescriber is uncertain about whether to prescribe an antibiotic for a suspected LRTI following clinical assessment, reflecting a more selective, targeted approach to POCT use."

Reviewer 3: Mr. Sajal Saha
 Comment 1: The rationale of the sample size for delphi pannel can be explained further? Authors may follow this work. "Manyara AM, Purvis A, Ciani O, Collins GS, Taylor RS. Sample size in multistakeholder Delphi surveys: at what minimum sample size do replicability of results stabilize?. Journal of Clinical Epidemiology. 2024 Oct 1;174:111485."
 Response 1: We appreciate this helpful suggestion. We have revised the Limitations section to acknowledge that recent empirical work by Manyara et al. (2024) suggests higher sample sizes may be needed for greater replicability:
 Page 12: "Additionally, although response rates across Delphi rounds were consistent with the recommended range of 5-25 participants for optimal consensus,(44-50) attrition from 19 participants in the first round to 12 in the final round could have affected the reliability of the final consensus. While the 12 remaining participants still represented a mix of healthcare professionals to provide diverse opinions, recent empirical work suggests that larger sample sizes may be required to achieve greater replicability of Delphi findings.(54) Manyara et al. recommend a minimum of 20-30 participants per stakeholder group to support moderate replicability in Delphi surveys, noting that smaller sample sizes (while common in the literature) may limit the stability of the results.(54) Our smaller panel reflects pragmatic challenges of recruiting busy clinicians in an emerging area of practice, but we acknowledge this as a methodological limitation."

Comment 2: The reliability of the responses in each round and overall could be measured if within the scope.

Response 2: Thank you for this thoughtful suggestion. We appreciate the importance of considering reliability in research design. However, we would like to clarify that within the context of a Delphi study, the primary objective is not to assess response reliability in the traditional sense, but rather to facilitate expert reflection and movement towards consensus through an iterative process. The Delphi method is specifically designed to allow participants to revise their views in light of anonymised group feedback provided between rounds. As such, changes in responses are not seen as inconsistencies or a lack of reliability, but as an intended and valuable outcome of the method. Applying conventional reliability measures, such as assessing response stability across rounds, would therefore be at odds with the core purpose of the Delphi process.

Comment 3: The key limitation of the paper is no involvement of GPs and community pharmacists who have used CRP POCT. The consensus statements could have been different if they were included. Authors can explain why there was no involvement of those GPs/community pharmacists?

Response 3: We appreciate this thoughtful observation. Our aim was to engage GPs and community pharmacists currently practising in Irish primary care, as they are the key stakeholders who would ultimately use CRP POCT if introduced into this healthcare system. Given that CRP POCT is not yet widely available in Ireland, it was not possible to include users with direct experience of the test. However, we intentionally sought the perspectives of these clinicians because they possess critical insight into how such technology could be integrated within Irish clinical workflows, reimbursement structures, and antimicrobial stewardship initiatives. We agree that the inclusion of experienced users, particularly from countries where CRP POCT is already embedded in practice, could provide valuable operational perspectives. We have expanded the Strengths and Limitations section to reflect this point and to recommend that future research incorporates input from such users during the validation and implementation phases. The revised text now reads:

Page 13: “It is also important to acknowledge that none of the panellists had prior experience using CRP POCT in practice, as the technology is not yet widely available in Irish primary care (Table 2). This likely influenced the perspectives captured and may have resulted in different results than if experienced users had participated. Future research should seek to validate and refine these guidance statements in real-world clinical settings, including input from healthcare professionals with hands-on experience using CRP POCT.”
